# Designer TALEs enable discovery of cell death-inducer genes

**DOI:** 10.1093/plphys/kiae230

**Published:** 2024-05-09

**Authors:** Roxana A Roeschlin, Sepideh M Azad, René P Grove, Ana Chuan, Lucila García, Regina Niñoles, Facundo Uviedo, Liara Villalobos, Maria E Massimino, María R Marano, Jens Boch, José Gadea

**Affiliations:** Instituto de Biología Molecular y Celular de Rosario (IBR)-Consejo Nacional de Investigaciones Científicas y Tecnológicas (CONICET), Ocampo y Esmeralda S/n, S2002LRK, Rosario, Argentina; Instituto de Biología Molecular y celular de Plantas (IBMCP), Universidad Politécnica de Valencia-CSIC, Ingeniero Fausto Elio S/N., 46022, Valencia, España; Institute of Plant Genetics, Leibniz Universität Hannover, 30419 Hannover, Germany; Instituto de Biología Molecular y celular de Plantas (IBMCP), Universidad Politécnica de Valencia-CSIC, Ingeniero Fausto Elio S/N., 46022, Valencia, España; Instituto de Biología Molecular y Celular de Rosario (IBR)-Consejo Nacional de Investigaciones Científicas y Tecnológicas (CONICET), Ocampo y Esmeralda S/n, S2002LRK, Rosario, Argentina; Área Virología, Facultad de Ciencias Bioquímicas y Farmacéuticas, Universidad Nacional de Rosario (UNR), Suipacha 590, S2002LRK, Rosario, Argentina; Instituto de Biología Molecular y celular de Plantas (IBMCP), Universidad Politécnica de Valencia-CSIC, Ingeniero Fausto Elio S/N., 46022, Valencia, España; Instituto de Biología Molecular y Celular de Rosario (IBR)-Consejo Nacional de Investigaciones Científicas y Tecnológicas (CONICET), Ocampo y Esmeralda S/n, S2002LRK, Rosario, Argentina; Instituto de Biología Molecular y Celular de Rosario (IBR)-Consejo Nacional de Investigaciones Científicas y Tecnológicas (CONICET), Ocampo y Esmeralda S/n, S2002LRK, Rosario, Argentina; Instituto de Biología Molecular y celular de Plantas (IBMCP), Universidad Politécnica de Valencia-CSIC, Ingeniero Fausto Elio S/N., 46022, Valencia, España; Instituto de Biología Molecular y Celular de Rosario (IBR)-Consejo Nacional de Investigaciones Científicas y Tecnológicas (CONICET), Ocampo y Esmeralda S/n, S2002LRK, Rosario, Argentina; Área Virología, Facultad de Ciencias Bioquímicas y Farmacéuticas, Universidad Nacional de Rosario (UNR), Suipacha 590, S2002LRK, Rosario, Argentina; Institute of Plant Genetics, Leibniz Universität Hannover, 30419 Hannover, Germany; Instituto de Biología Molecular y celular de Plantas (IBMCP), Universidad Politécnica de Valencia-CSIC, Ingeniero Fausto Elio S/N., 46022, Valencia, España

## Abstract

Transcription activator-like effectors (TALEs) in plant-pathogenic *Xanthomonas* bacteria activate expression of plant genes and support infection or cause a resistance response. PthA4^AT^ is a TALE with a particularly short DNA-binding domain harboring only 7.5 repeats which triggers cell death in *Nicotiana benthamiana*; however, the genetic basis for this remains unknown. To identify possible target genes of PthA4^AT^ that mediate cell death in *N. benthamiana*, we exploited the modularity of TALEs to stepwise enhance their specificity and reduce potential target sites. Substitutions of individual repeats suggested that PthA4^AT^-dependent cell death is sequence specific. Stepwise addition of repeats to the C-terminal or N-terminal end of the repeat region narrowed the sequence requirements in promoters of target genes. Transcriptome profiling and *in silico* target prediction allowed the isolation of two cell death inducer genes, which encode a patatin-like protein and a bifunctional monodehydroascorbate reductase/carbonic anhydrase protein. These two proteins are not linked to known TALE-dependent resistance genes. Our results show that the aberrant expression of different endogenous plant genes can cause a cell death reaction, which supports the hypothesis that TALE-dependent executor resistance genes can originate from various plant processes. Our strategy further demonstrates the use of TALEs to scan genomes for genes triggering cell death and other relevant phenotypes.

## Teaser

The modularity of transcription activator-like effectors enables regulating their specificity and potential target sites, revealing two cell death inducer genes in *Nicotiana benthamiana*.

## Introduction

The hypersensitive response (HR) is a form of programmed cell death in plants that effectively prevents pathogen infection by sacrificing cells in the infected area. It is generally triggered upon recognition of virulence proteins called effectors from the pathogen (Toruño et al. 2016; Thordal-Christensen 2020). Often, resistance (R) proteins recognize these effectors once delivered into the cytoplasm of the plant cell by the pathogen, via the type III secretion system (T3SS) (Büttner and He 2009). Typically, these R proteins are cytoplasmic proteins possessing both a nucleotide-binding site (NBS) and leucine-rich (LRR) repeat domains known as NBS–LRR proteins or NLRs (Kourelis and Van Der Hoorn 2018; Tamborski and Krasileva 2020).

Transcription activator-like effectors (TALEs) are proteins from *Xanthomonas* pathogens that function as plant transcription factors. After being delivered into plant cells by the T3SS, TALEs are transported into the nucleus, where they specifically match with effector-binding elements (EBEs) present in host promoters, leading to the activation of target genes (Boch and Bonas 2010; Timilsina et al. 2020). In this way, native TALEs evolved different target specificities to induce the expression of different genes (termed susceptibility genes) supporting bacterial infection (Cernadas et al. 2014; Hu et al. 2014; Erkes et al. 2017; Schwartz et al. 2017; Mücke et al. 2019; Teper and Wang 2021; Zhang et al. 2022). Functional TALE domains comprise an N-terminal domain for T3SS secretion and translocation, a central DNA-binding domain, and a C-terminal domain with nuclear localization signals (NLSs) and a eukaryotic acidic transcriptional activation domain (Boch and Bonas 2010). The modular DNA-binding domain consists of tandem 34 amino acid repeats, each one binding to a single DNA nucleotide. Specificity is determined by two variable amino acids at positions 12 and 13 termed repeat-variable diresidue (RVD) (Boch et al. 2009; Moscou and Bogdanove 2009). In addition, the N-terminal region next to the first repeat recognizes an additional nucleotide, mainly thymine, though other nucleotide types at this “zeroth” position have been demonstrated to allow binding in certain contexts (Yu et al. 2011; Doyle et al. 2013; Schreiber and Bonas 2014). The last repeat is only conserved for 20 amino acids and therefore termed a half repeat. Thus, a simple “TALE code” enables to predict the optimal DNA target sequence from the sequence of RVDs within the TALE array (Boch et al. 2009; Moscou and Bogdanove 2009). In addition, the modular DNA-binding domain allows a simple rearranging of repeats to build designer TALEs with any desired DNA-binding specificity (Cermak et al. 2011; Geißler et al. 2011; Weber et al. 2011). Accordingly, TALEs have been employed as gene activators in synthetic biology (Li et al. 2015; Schreiber and Tissier 2017; Schreiber et al. 2019) and started the genome editing revolution as TALE-nuclease fusions (Christian et al. 2010; Miller et al. 2010; Li et al. 2011; Kim et al. 2015; Becker et al. 2022).

Plants can recognize TALEs and deploy a battery of defenses ultimately culminating in an HR to thwart pathogen colonization. Two different mechanisms for TALE recognition have been described. One occurs in the cytoplasm and involves NLR proteins which perceive TALE topology, irrespective of their DNA-binding specificity. For example, the tomato (*Solanum lycopersicum*) NLR protein Bs4 mediates recognition of both AvrBs3 and AvrBs4, two TALEs sharing 96% sequence identity but having distinct DNA-binding specificities (Boch et al. 2009). The other mechanism involves executor resistance genes (ex-*R*), which act as traps for TALEs and trigger cell death upon expression, thereby effectively blocking bacterial infection (Zhang et al. 2015; Nowack et al. 2022). Up to now, only six ex-*R* genes associated with the corresponding TALEs have been cloned: *Bs3* and *Bs4C-R* from pepper and *Xa7*, *Xa10*, *Xa23*, and *Xa27* from rice (Gu et al. 2005; Römer et al. 2009; Strauß et al. 2012; Tian et al. 2014; Wang et al. 2015; Chen et al. 2021; Luo et al. 2021). Besides their functional similarity to induce cell death, only *Xa10* and *Xa23* are similar on the protein level, suggesting that many different ways exist among ex-*R* genes to initiate cell death (Zhang et al. 2015; Nowack et al. 2022). In general, the molecular details of how these ex-*R* genes signal the cell to die are still unknown. The lack of homology between ex-*R* and NLR genes demonstrates that their initial mechanism is different, although they might eventually trigger related effects, like ion fluxes across membranes, in an example of convergent evolution (Balint-Kurti 2019).

Recently, we identified an unusually short TALE, named PthA4^AT^, in a variant of *Xanthomonas citri* subsp. *citri* (*X. citri*) named *X. citri A^T^*. This TALE harbors only 7.5 repeats and is both necessary and sufficient to trigger an HR in *Citrus limon* and to confer resistance to citrus canker (Roeschlin et al. 2017; Roeschlin et al. 2019). PthA4^AT^ also triggers cell death when infiltrated via *Agrobacterium* in *Nicotiana benthamiana* (Roeschlin et al. 2019). Disrupting the three NLSs of PthA4^AT^ by point mutations impairs the development of this response, indicating that nuclear localization is required. Moreover, PthA4^AT^ is able to mediate transcriptional activation, and the HR is greatly attenuated when the C-terminal domain containing the activation domain is removed, pointing to a mechanism for PthA4^AT^ perception operating in both *C. limon* and *N. benthamiana* based on host gene expression (Roeschlin et al. 2019).

A TALE with such a short DNA target sequence is expected to find multiple EBEs within the promoters of all *Nicotiana* genes. Different bioinformatic approaches have been developed to predict potential target sites of TALEs in genomic sequences according to RVD specificities (Doyle et al. 2012; Grau et al. 2013; Pérez-Quintero et al. 2013; Erkes et al. 2019, 2021). These prediction tools often yield numerous putative targets, which requires further experimental evidence of gene activation to identify genes triggering a specific phenotype (Boch et al. 2014).

Here, we employ designer TALEs in an innovative strategy to reduce the number of potential target sites and identify plant genes responsible for the PthA4^AT^-induced cell death in *N. benthamiana*. Our results reveal that some *R* genes are in principle not linked to immunity-related functions, revealing pathways leading to cell death activation in plants.

## Results

### PthA4^AT^ blocks disease progression of *Xanthomonas campestris* pv. *campestris* (Xcc8004) and tobacco necrosis virus in *N. benthamiana*

**Figure 1.**
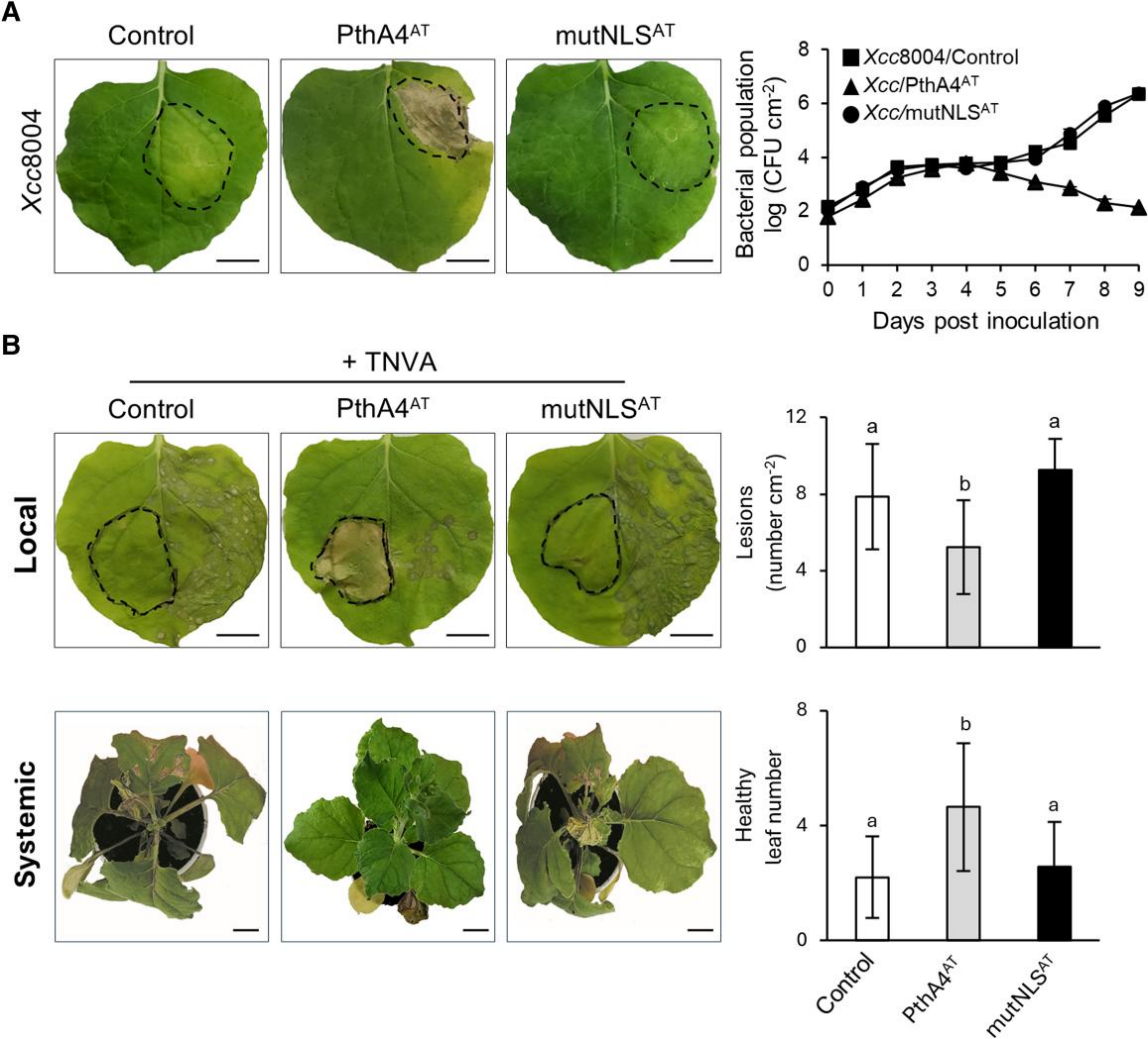
PthA4^AT^ confers protection against *X. campestris* pv. *campestris* (*Xcc*8004) and TNVA in *N. benthamiana* leaves. **A)** Macroscopic symptoms observed on leaves inoculated by infiltration with *Xcc*8004 harboring PthA4^AT^ or its derivative mutated in the three NLSs (mutNLS^AT^) at 5 dpi. Bacterial growth on *N. benthamiana* leaves (CFU per cm^2^) after inoculation along 9 dpi. Values are the mean ±SD of three independent biological replicates. Control: *Xcc*8004 transformed with empty vector. **B)** Necrotic lesions observed at 3 dpi on leaves rub-inoculated with TNVA. *N. benthamiana* leaves were infiltrated via *Agrobacterium* transformed with PthA4^AT^ or mutNLS^AT^ on one half of the leaf, and the other half was inoculated with TNVA 4 days later. Upper panel: Local protection of PthA4^AT^ was measured as the number of lesions per cm^2^ at 3 dpi using ImageJ software v1.4. Lower panel: Systemic protection was measured as the number of emerging leaves without necrosis (healthy leaf number) at 12 dpi. Values are expressed as means ± SD of three independent biological replicates. Letters indicate significant differences at *P*-value < 0.01 (Tukey's test). Control: *Agrobacterium* transformed with empty vector. Images were digitally extracted for comparison. Dashed lines indicate infiltrated area. Scale bar: 20 mm.

Previously, agroinfiltration assays demonstrated that nuclear localization of *X. citri* TALE PthA4^AT^ triggered cell death in *N. benthamiana* (Roeschlin et al. 2019). We investigated whether this response may hinder the progress of pathogenic bacteria and viruses. Infiltration with *Xanthomonas campestris* pv. *campestris* strain 8004 (*Xcc*8004) causes macroscopic chlorosis coupled with bacterial growth in *N. benthamiana* (Yun et al. 2006; Fig. 1A). In contrast, *Xcc*8004 harboring PthA4^AT^ (*Xcc*/PthA4^AT^) triggered a cell death response and halted disease progression, leading to a substantial reduction of *Xcc*8004 bacterial population in the infiltrated area (Fig. 1A). Similar to *Citrus* (Roeschlin et al. 2019), PthA4^AT^ is translocated into the *N. benthamiana* nucleus (Supplementary Fig. S1), and the arrest of bacterial progression was not observed in leaves inoculated with an *Xcc*8004 expressing the PthA4^AT^ derivative mutated in the NLSs (mutNLS^AT^) (Fig. 1A).

Similar results were obtained using a different plant–pathogen interaction. Agroinfiltration of PthA4^AT^ into one half of the *N. benthamiana* leaf and subsequent rub inoculation with tobacco necrosis virus A (TNVA) into the neighboring half significantly reduced the number of virus-induced necrotic lesions, whereas using the mutNLS^AT^ variant did not (Fig. 1B). Furthermore, the number of distal leaves with necrosis symptoms also decreased in PthA4^AT^-infiltrated plants relative to the controls (Fig. 1B). Taken together, these findings indicate that PthA4^AT^ induces a cell death response that effectively impedes the disease progression of pathogenic bacteria *Xcc*8004 and TNVA virus in *N. benthamiana*.

### PthA4^AT^-mediated cell death is dependent on specific RVDs

**Figure 2.**
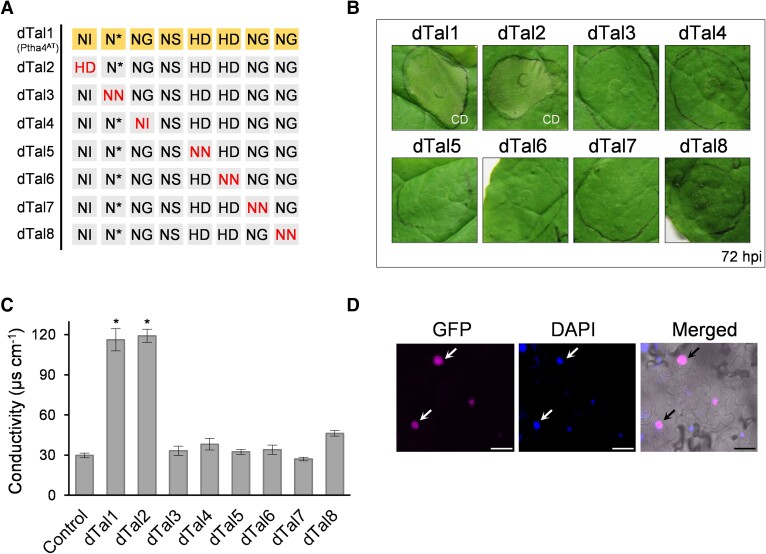
Substitution of individual RVDs of dTal1/PthA4^AT^. **A)** RVDs of dTALEs where PthA4^AT^-RVDs have been modified to target a different base. RVDs of dTal1 (PthA4^AT^) are shown in the first row. Red letters (HD in first column in dTal2; NN in second column in dTal3; NI in third column in dTal4; NN in fifth column in dTal5; NN in sixth column in dTal6; NN in seventh column in dTal7; NN in last column in dTal8) indicate RVD substitutions. **B)** Cell death development on *N. benthamiana* leaves infiltrated with the different constructs shown in A and photographed 72 hpi.**C)** Cell damage evaluated by conductivity measurement at 72 hpi. Values are the mean ± SD of three independent biological replicates. * significant compared to control (one-way ANOVA, Tukey's test, *P*-value < 0.01). Control: infiltration medium. **D)** Representative image of GFP (magenta) and DAPI (blue) signal in the nucleus (arrows) of *N. benthamiana* leaves expressing a GFP-dTALE fusion at 72 hpi. Left: fluorescence image; right: merged image (bright field). Scale bar: 40 *µ*m. CD, cell death.

To test whether PthA4^AT^ triggers cell death through the expression of one or several specific host genes, we exploited the modularity of the TALE DNA-binding specificity and engineered designer dTALEs (named dTals) with a controlled RVD composition (Geißler et al. 2011). The four most common RVDs in nature (NI, HD, NN, and NG) specify bases A, C, G/A, and T, respectively. Less common RVDs such as N* can target C/T, and NH can achieve guanine-specific recognition. NS, however, can target any of the four bases (Cong et al. 2012; Streubel et al. 2012). Initially, a designer TALE (dTal1) containing the same RVDs as PthA4^AT^ was engineered. Similar to PthA4^AT^, dTal1 triggers cell death in the infiltrated area at 72  hours post-inoculation (hpi) and confers resistance to TNVA (Supplementary Fig. S2). Next, we generated seven dTALEs (dTal2 to dTal8) to substitute individual RVDs throughout dTal1 with RVDs targeting a different base, thereby altering their target specificity (Fig. 2A). Only dTal2, where NI was substituted by HD in the first repeat, was able to induce cell death at 72 hpi (Fig. 2B). To quantify this response, we measured ion leakage resulting from membrane damage. Conductivity was increased only in the tissue expressing dTal1 and dTal2, confirming that these dTALEs triggered a cell death response (Fig. 2C). GFP signals were detected within the nucleus for all dTALEs, indicating their proper expression and translocation (Fig. 2D).

Additional experiments using the same strategy for target refinement of RVDs in positions two (N*) and four (NS) further suggest a sequence dependence for PthA4^AT^-mediated HR development, although two-base specificities are possible for position four (Supplementary Fig. S3). Taken together, our results demonstrate that re-targeting PthA4^AT^ to alternative recognition sites by substituting specific RVDs disrupts cell death activation, suggesting that PthA4^AT^ triggers cell death by one or several specific host genes. Our data also suggest that the designer TALE technology is a promising tool to identify cell death inducer genes in *N. benthamiana* using PthA4^AT^ as a starting point.

### Stepwise extension of the RVD domain at the C- and N-terminal ends refines specificity for PthA4^AT^-mediated cell death activation

**Figure 3.**
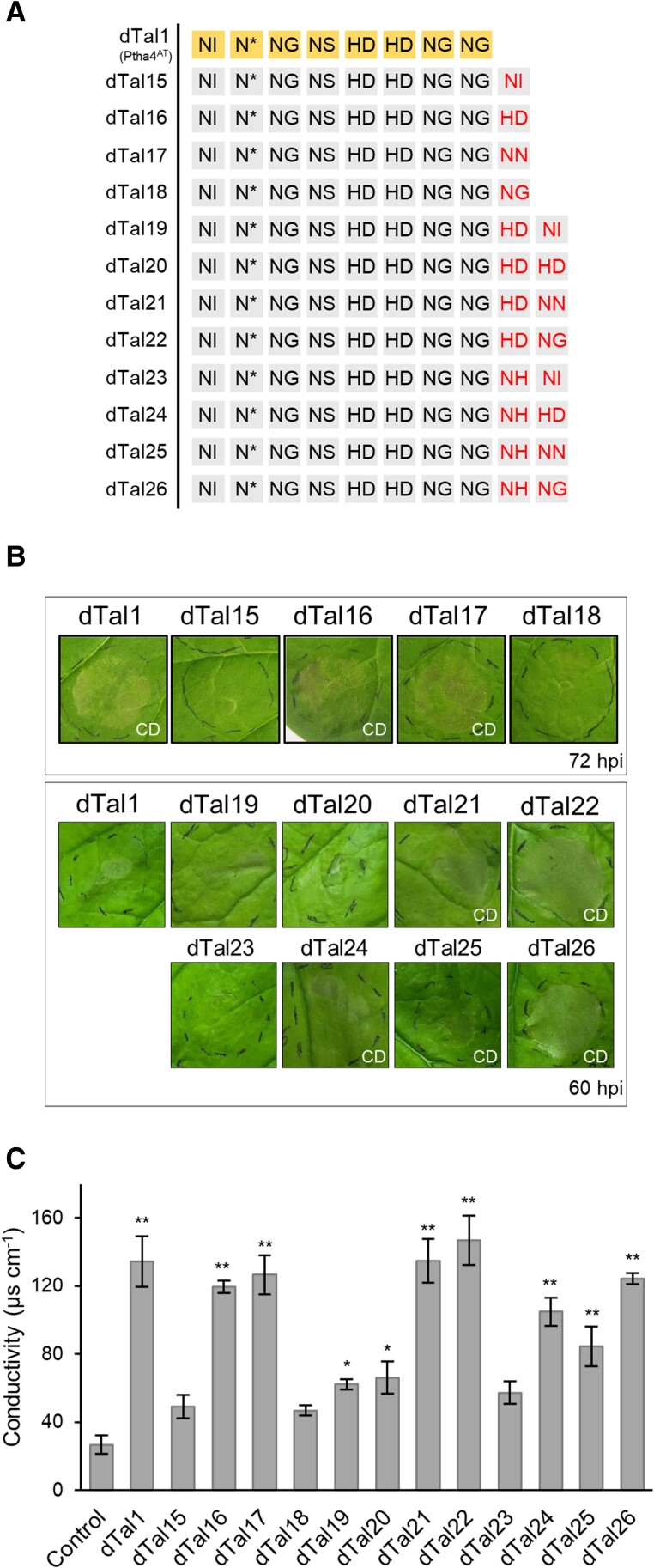
Extensions on the C-terminal end of the repeat domain of dTal1. **A)** RVDs of the different dTALEs. RVDs of dTal1 (PthA4^AT^) are shown on yellow background in the first row. Red letters in the last two columns indicate RVD extensions. **B)** Cell death response on leaves infiltrated via *Agrobacterium* photographed at 72 hpi. **C)** Cell death quantification by conductivity measurements at 72 hpi. Values are the mean ± SD of three independent biological replicates. **: significant compared to control (one-way ANOVA, Tukey's test, *P*-value < 0.01). Control: infiltration medium. CD, cell death.

PthA4^AT^ is predicted to bind to a target sequence of 9 bp in promoter regions (8 bp via the RVD region plus the preceding base, Fig. 2A), resulting in the activation of target genes. This short sequence will occur frequently by chance in the *N. benthamiana* genome. As the number of candidate genes is expected to decrease with an increasing number of RVDs, additional PthA4^AT^ derivatives with additional repeats were built that specify longer target DNA sequences. This should eventually facilitate the identification of those genes responsible for the cell death phenotype. Individual repeats were added to the C-terminal end of the dTal1 repeat region to specify either A, C, G, or T. The ability of these longer dTALEs to still trigger cell death was then assayed in *N. benthamiana*. dTal15, dTal16, dTal17, and dTal18 added NI, HD, NN, or NG to dTal1 (Fig. 3A). Infiltration of dTal16 and dTal17, but not of dTal15 and dTal18, resulted in cell death at 72 hpi (Fig. 3B). These observations were confirmed by conductivity measurements (Fig. 3C). dTal16 and dTal17 were then used for another round of extension. dTal16 was extended by one repeat, resulting in dTal19, dTal20, dTal21, and dTal22. A modified version of dTal17 (with NN in its last position substituted by NH, to confer better specificity for G) was also extended, resulting in dTal23, dTal24, dTal25, and dTal26 (Fig. 3A). Cell death was observed for dTal21 and dTal22 and also for dTal24, dTal25, and dTal26 at 60 hpi, earlier than dTal1-induced cell death (Fig. 3B). Base preference for T in position two was confirmed in this longer RVD context (Supplementary Fig. S4). Quantitative differences were confirmed by electrolyte leakage measurements (Fig. 3C). We noticed that PthA4^AT^ derivatives with different RVD combinations activate cell death. This indicates either that some RVD-base mismatches are tolerated or that these dTALEs target different cell death inducer genes.

We wondered if it is also possible to add repeats to the N-terminal end of the repeat region, because the N-terminal repeats have a higher impact on the overall DNA-binding capability of TALEs than C-terminal ones (Meckler et al. 2013). Nevertheless, extending at the N-terminal end is not straightforward, because the N-terminal domain of TALEs typically specifies a T preceding the target sequence of the repeat region (Boch et al. 2009). To allow extension of our dTALEs on the N-terminal end of the repeat region, we used an N-terminal domain which can recognize all four bases (Lamb et al. 2013). Using this, we extended dTal27 (10 RVDs) which causes a strong cell death, by two N-terminal repeats specifying AT, CT, GT, and TT, respectively. Following infiltration, the dTALE specifying TT (dTal60) caused a strong cell death, while AT- or CT-specifying dTALEs (dTal57 and dTal58, respectably) caused an intermediate and the dTALE specifying GT (dTal59) only a low reaction (Supplementary Fig. S5). A further round of N-terminal extension of one repeat specified for TTT and GTT causing the strongest cell death (dTals 63 and 64; Supplementary Fig. S5). Taken together, these results show that stepwise addition of repeats to the C- and N-terminal ends of the RVD region results in dTALEs with longer target sequences that show a differential cell death induction at individual target sites.

### Transcriptomic profiling and TALE target prediction identify *PAT1* as a cell death inducer gene in *N. benthamiana*

**Figure 4.**
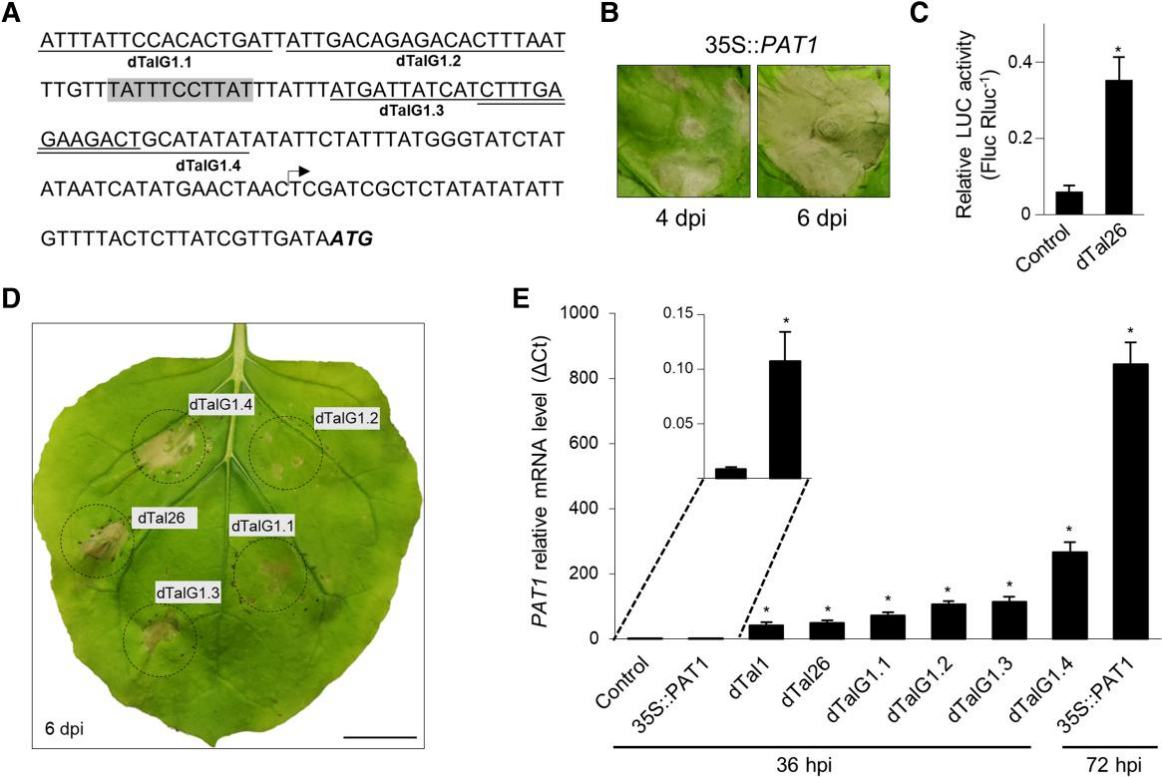
Cell death activation by patatin-like protein PAT1. **A)***PAT1* promoter sequence showing the binding site of different dTALEs. Arrow, TSS. Start codon ATG is shown in bold and italics. The predicted dTal26-binding site is shown in gray. *PAT1*-specific designer dTALEs are underlined. **B)** Cell death response in *N. benthamiana* leaves infiltrated via *Agrobacterium* containing the *PAT1* cDNA under the control of the CaMV35S promoter. Pictures were taken at 6 and 8 dpi. **C)** Relative luciferase activities of LUC (luciferase) reporter under the control of the *PAT1* promoter (*pPAT1::LUC*) in *N. benthamiana* leaves co-infiltrated with dTal26 or with *Agrobacterium* transformed with empty vector (Control). Data are presented as Fluc Rluc-1 ± SD. * significant after Tukey's test (*P*-value < 0.05). Three biological replicates were performed. Each replicate consists of one 8-mm-diameter disc per infiltrated leaf collected at 36 hpi. **D)** Cell death response on *N. benthamiana* leaves infiltrated with *PAT1*-dTALEs constructs and photographed at 6 dpi. Images were digitally extracted for comparison. Black circles delimit infiltrated regions. Dashed lines: accurate infiltrated area. Scale bar: 20 mm. **E)***PAT1* mRNA levels analyzed by RT-qPCR at 36 and 72 hpi. Results are presented as mean ± SD. *, significant after *t* test (*P*-value < 0.005). Control: infiltration medium. Three replicates per sample were performed.

Target genes can be identified by analyzing plant transcripts that are activated in the presence of specific TALEs, in combination with *in silico* prediction of TALE target sequences in promoter regions (Hu et al. 2014; Schwartz et al. 2017). dTal26 was used to identify cell death inducer genes. Similar to dTal1 and PthA4^AT^, infiltration via *Agrobacterium* of dTal26 to *Nicotiana* leaves protects against subsequent infection by TNVA virus (Supplementary Fig. S6). dTal26 triggers cell death faster than dTal1, covering the whole infiltrated area at 60 hpi (Fig. 3B), suggesting that extending the binding site confers better ability to induce gene expression of cell death inducer genes. RNA of *N. benthamiana* leaves infiltrated with *Agrobacterium* delivering dTal26 was sampled at 36 hpi, when the effector was expressed but cell death was not yet visible (Supplementary Fig. S7). A dTALE that does not trigger cell death was used as a negative control. To predict dTal26-binding sites, promoter regions spanning 400 nt upstream to 200 nt downstream of the transcriptional start site from dTal26-induced genes were obtained from the *N. benthamiana* genome, and PrediTALE (Erkes et al. 2019) was used (with dTal26 RVDs as input and N* substituted by NG in the second position, to refine the actual requirement for T at this position, Supplementary Fig. S4). Although longer than PthA4^AT^, the relatively short length of dTal26 posed a challenge due to its low binding specificity (no target with *P*-value < 10^−6^, Supplementary Table S1), hindering the filtering of candidates based on robust DNA-binding predictions. Nonetheless, applying stringent expression criteria for target prioritization identified 34 genes (Supplementary Table S1). Out of these, five genes fulfilled the promoter sequence requirements at the 5′ end of dTal26-predicted binding site for a cell death inducer gene, as outlined in the previous section, and were selected for overexpression (Supplementary Fig. S8 and Fig. 4A). Of these, only the NBlab18G05420 gene, encoding the patatin-like protein PAT1, showed a cell death response at 4 days post-inoculation (dpi) when overexpressed in *N. benthamiana* (Fig. 4B), suggesting a role as a cell death inducer gene.

### *PAT1* expression is not sufficient to trigger the entire dTal26-mediated cell death response

The involvement and dynamics of PAT1 in cell death activation was studied through several experimental approaches. First, a dual-luciferase reporter assay was used to test activation of *PAT1* by dTal26. A promoter region of *PAT1*, containing the predicted target sequence for dTal26, was cloned to direct expression of luciferase (*pPAT1::LUC*). Co-expression of dTal26 and *pPAT1*::*LUC* by agroinfiltration demonstrated that dTal26 is able to induce *PAT1* expression 5-fold at 36 hpi (Fig. 4C). Next, four dTALEs targeting the *PAT1* promoter were generated (Fig. 4A). Agroinfiltration, delivering each one of them individually, induced cell death visible at 6 dpi (Fig. 4D), confirming that TALE-mediated induction of *PAT1* triggers cell death. Notably, *PAT1*-induced cell death is visible at later times than that triggered by dTal26. Consequently, we investigated the levels of *PAT1* expression under the different activation conditions. Expression of *PAT1* increases at 36 hpi after delivering dTal1 or dTal26 (Fig. 4E), confirming RNA-seq data. Similar levels were observed at 36 hpi after infiltration with *PAT1*-specific dTALEs, although cell death was observed much later. Levels of expression were low at 36 hpi after agroinfiltration with *35S::PAT1* (Fig. 4E inset), but high at 72 hpi, even though cell death will not be visible yet (Fig. 4B), suggesting that the strong dTal26-mediated cell death is likely caused by a combination of *PAT1* and additional genes.

### PthA4^AT^-extended dTALEs with 14 RVDs retain the cell death phenotype and allow the identification of an additional cell death inducer gene

**Figure 5.**
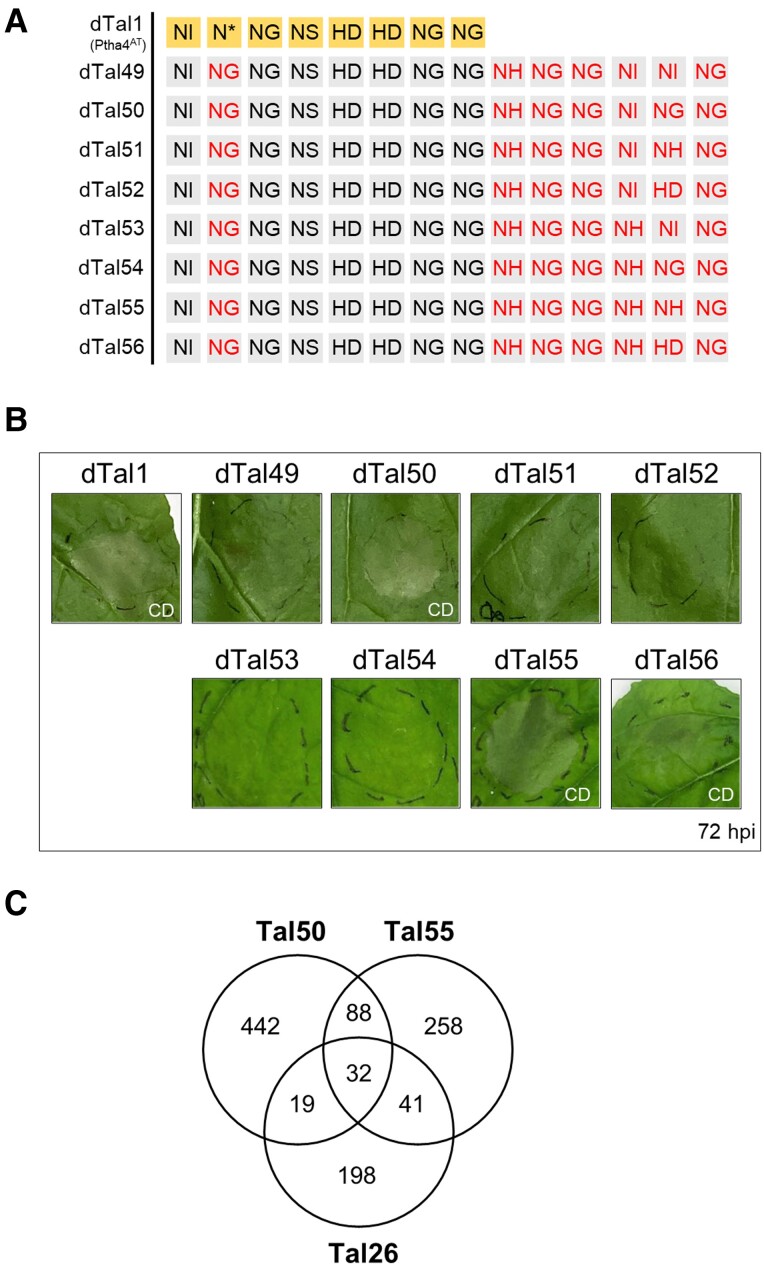
Extensions of six repeats on the C-terminal end of the RVD domain of dTal1. **A)** RVDs of the different dTALEs derived from dTal46 and dTal47. RVDs of dTal1 (PthA4^AT^) are shown on yellow background in the first row. Red letters indicate RVD substitutions (NG in second column in all dTals) or extensions (six last columns). **B)** Cell death response on *N. benthamiana* leaves infiltrated via *Agrobacterium* Pictures were taken at 72 hpi. **C)** Venn diagram of induced genes in *N. benthamiana* leaves infiltrated with dTal26, dTal50, or dTal55 and sampled at 36 hpi (Log_2_FoldChange > 1; adjusted *P*-value < 0.05) combined with PrediTALE predictions (*P* < 10^−5^). CD, cell death.

We aimed to identify additional cell death inducer genes that are triggered by PthA4^AT^ besides *PAT1*. To further reduce the number of predicted targets, we added repeats C-terminally to the dTal27 repeats (10 RVDs) to make the dTALE more specific. Following the same strategy as above, additional dTALEs were constructed and assayed for cell death response after infiltration. (Supplementary Fig. S9 and Fig. 5A). These experiments revealed four 14 RVD-long dTALEs still capable of developing cell death (Fig. 5B). To search for the corresponding cell death inducer genes, we performed a second transcriptomic assay to obtain genes induced by two of them (dTal50 and dTal55). The expression data were combined with PrediTALE results for binding prediction to get a new list of putative targets. To avoid candidates being discarded during the filtering process, more relaxed expression criteria than those used in the dTal26 analysis above were applied. In this new analysis, 290 genes were considered putative targets of dTal26, 580 of dTal50, and 419 of dTal55. Among them, 32 genes were predicted targets of all three dTALEs (Fig. 5C), whereas 60 genes were predicted targets of dTal26 and only one of the two other dTALE derivatives. Twenty-seven of these last 61 genes, however, contain a binding site for both dTALEs (dTal50 and dTal55) in its promoter region when considering a *P*-value 10^−4^ in PrediTALE, suggesting that they could also be targets for the three dTALEs. A list of 92 genes was generated of potential cell death inducer genes (Supplementary Table S2).

**Figure 6.**
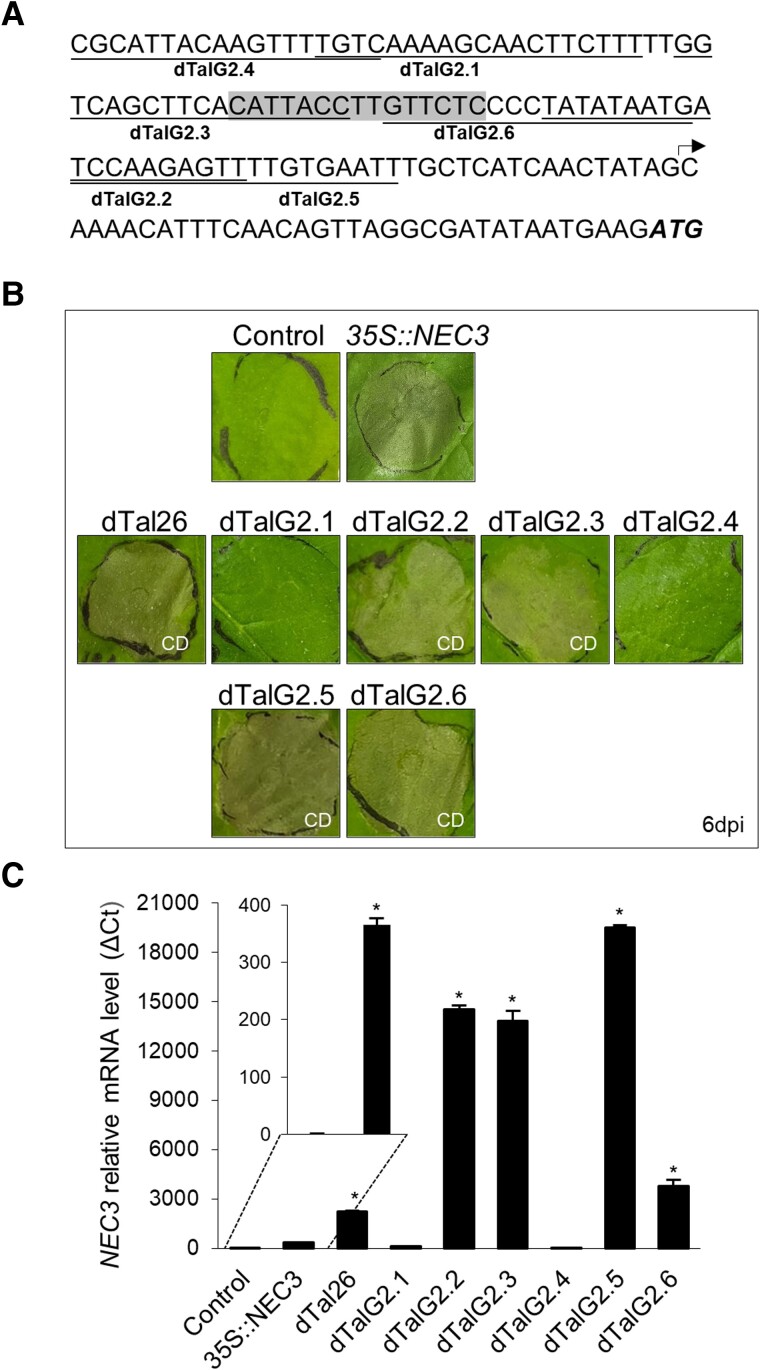
NBlab07G09180, encoding a bifunctional monodehydroascorbate reductase and carbonic anhydrase nectarin-3 (NEC3), mediates cell death induction in *N. benthamiana*. **A)** Graphical representation of the *NEC3* promoter region. Gene-specific dTALEs (dTalG2.1 to dTalG2.6) are underlined. dTal26/dTal50/dTal55 effector-binding element is highlighted in gray. Arrow indicates TSS. The start codon (ATG) is shown in bold italics. **B)** Cell death response on leaves infiltrated via *Agrobacterium* with 35S::*NEC3* and gene-specific dTALEs. Pictures were taken at 6 dpi.**C)***NEC3* mRNA levels analyzed by RT-qPCR at 48 hpi for dTALEs and 72 hpi for 35S::*NEC3*. Results are presented as mean ± SD. * significant after *t* test (*P*-value < 0.005). Control: infiltration medium. Three replicates per sample were performed. CD, cell death.

We selected genes from this list (based on gene function, expression data, and sequence motifs) and engineered dTALEs targeting their promoters to test them individually for cell death induction in *N. benthamiana.* Four dTALEs designed to activate the NBlab07G09180 gene, encoding a possible bifunctional monodehydroascorbate reductase and carbonic anhydrase nectarin-3 (NEC3), were able to activate the gene and trigger cell death (Fig. 6, A, B and C). In addition, overexpression of the NBlab07G09180 gene using a 35S promoter fusion also triggered cell death (Fig. 6B), indicating that the *NEC3* gene is another cell death inducer gene in *N. benthamiana*.

## Discussion

The modularity of TALEs opens the possibility to use them as probes for screening strategies, where the endogenous activation of specific genes by a given TALE translates into a visible phenotype. In a strategy starting from less stringent short TALEs, longer dTALEs with increased target specificity are engineered by stepwise extension of the RVD domain and those still retaining the phenotype of interest are selected (Supplementary Fig. S10). A classical strategy used to identify TALE target genes, based on the combination of global gene expression and binding-site prediction, is then used to identify and validate genes. This strategy can now be used for other screening strategies where a visible phenotype can be tracked.

In this study, we exploited this strategy to identify target genes involved in cell death activation in *N. benthamiana* by the short TALE PthA4^AT^ (Roeschlin et al. 2019). PthA4^AT^, a natural TALE triggering HR in *C. limon* (Roeschlin et al. 2017), also triggers cell death via ex-*R* genes when ectopically expressed in *N. benthamiana* (Roeschlin et al. 2019). Moreover, the possibility of PthA4^AT^-mediated cell death being triggered after recognition of the effector in the cytoplasm in a Bs4-like matter is discarded, as disrupting the three NLSs or removing the activation domain impairs the development of this response. This experimental system was exploited here as a proof of concept to identify additional genes regulating this biological process. We could clarify that PthA4^AT^ causes cell death via expression of specific genes. Furthermore, this work supports the concept that genes involved in normal physiological processes can cause cell death when their expression is misregulated by a TALE, which is significant for understanding ex-*R* gene evolution and developing novel plant resistances.

Up to date, six cell death inducer genes, induced by natural TALEs in specific plant–pathogen interactions, have been identified which function as *R* genes (Gu et al. 2005; Römer et al. 2009; Strauß et al. 2012; Tian et al. 2014; Wang et al. 2015; Chen et al. 2021; Luo et al. 2021). PthA4^AT^ is an unusual TALE with only 7.5 repeats. Short TALEs with few repeats will statistically bind to more target sites in a genome than longer ones with more repeats, which in principle increases their potential as discovery tools. However, this also increases the possibility of these short TALEs binding to multiple genes and generating an unspecific cytotoxic effect. This does not seem to be the case for PthA4^AT^. Despite harboring only 7.5 repeats, the strong cell death visible at 72 hpi is disrupted or delayed when the RVD composition is modified, indicating that it is the induced expression of specific target genes which causes cell death. The short length of PthA4^AT^ imposed, however, a difficulty to the identification of these host genes, beyond the degeneracy of the code for some RVDs and the mismatch tolerance common to all TALEs (Meckler et al. 2013; Rogers et al. 2015; Rinaldi et al. 2017). The high number of putative target genes in the *Nicotiana* genome did not allow a computational prediction of the one/s responsible for the cell death response. We approached this problem by stepwise extensions of the repeat domain of PthA4^AT^ either on the C-terminal or N-terminal side. This strategy allowed to generate longer PthA4^AT^ derivatives extended up to six repeats C-terminally or three repeats N-terminally that still were able to trigger cell death, while significantly reducing the number of possible targets.

The hypothesis of PthA4^AT^ activating more than one executor gene became apparent as our study advanced. This possibility was initially revealed when the substitution of the ambiguous NS by other RVDs with affinities for individual bases identified two possibilities for this position to trigger cell death. Eventually, we analyzed the transcriptional response to three different designer TALEs (dTal26, dTal50, dTal55) and identified a cell death inducer genes (NBlab07G09180, encoding a possible bifunctional monodehydroascorbate reductase and NEC3) that was induced by all three TALEs and an additional one (NBlab15G13810, encoding a patatin-like proteins) that was a predicted target of dTal26, but only of one of its two longer derivatives (dTal55). This confirmed that PthA4^AT^ and the shorter dTALEs targeted more than one cell death inducer gene. Accordingly, the cell death response observed at 72 hpi for dTal26 was stronger than that observed at the same timepoint for the longer dTal50 and dTal55 or any of the identified cell death inducer genes themselves. Taken together, our results indicate that there is likely an additive effect with the contribution of several high-effect and low-effect executor genes activated following PthA4^AT^ inoculation.

Our data support the recently postulated hypothesis that some TALE-induced ex-*R* genes may not originally function in plant defense (Nowack et al. 2022). Instead, the presence of TALE target sites in their promoter might have possibly occurred by chance, and such plants were further selected by humans because of their pathogen resistance. This is nicely exemplified by the *Xa23* rice resistance gene, functional against the TALE AvrXa23 from *Xanthomonas oryzae* pv. *oryzae*. Only in the resistant rice cultivar, a transposon and the AvrXa23 target sequence are inserted into the promoter of a small open reading frame, encoding a possible transmembrane protein (Wang et al. 2015). XA23 is similar to XA10, which also functions as an executor, involved in calcium transport across the endoplasmic reticulum membrane (Tian et al. 2014; Wang et al. 2015). The physiological functions of Xa10 and Xa23 are unknown, but it is plausible that they still contain, or originally contained, one that is distinct from their role as resistance gene. The function of our identified targets as genes participating in normal cellular processes, unrelated to classical immune responses, reinforces the view that executor genes are primarily involved in cellular processes including developmental programmed cell death and inadvertently trigger uncontrolled cell death when activated by an external TALE (Nowack et al. 2022). Patatin-like proteins are phospholipases involved in lipid peroxidation and represent the major storage protein in potato tubers. Intriguingly, patatin-like proteins are also induced in plant–*Xanthomonas* interactions (Cacas et al. 2009; Kim et al. 2014). Nectarins are proteins from the floral nectar. NEC3 is a major constituent of ornamental *Nicotiana* nectar and was found to contain carbonic anhydrase and monodehydroascorbate reductase activities (Carter and Thornburg 2004). A biological interpretation for any of the two candidates is speculative at this point. In summary, the targets identified in this study shed light on distinct cellular processes that can cause cell death.

To our knowledge, PthA4^AT^ is the only 7.5 RVDs TALE with a biological function. In TALEs of this length, transcriptional activation is still occurring (Boch et al. 2009; Roeschlin et al. 2019). The strain containing PthA4^AT^ is still infective in the host from which it was isolated (*Citrus clementina*), and cell death was observed only in other *Citrus* hosts (*C. limon* and *Citrus sinensis*) (Chiesa et al. 2013; Roeschlin et al 2019). The transcriptional program triggered by PthA4^AT^ in *C. limon* resembles an immune response and protects the plant from future bacterial infections (Roeschlin et al. 2017), and we showed here that the cell death response in *N. benthamiana* also results in plant protection to subsequent attacks of bacteria and viruses. This suggests that physiological cell death reactions could reconstitute a functional pathogen resistance and unveil avenues for leveraging cell death inducer genes to engineer resistances in economically pivotal crops. Noteworthy, the overexpression of patatin-like genes in Arabidopsis shows cell death responses (La Camera et al. 2009) and conferred resistance to *Pseudomonas syringae* pv. *tomato* (Pst) and *Hyaloperonospora arabidopsidis* infection (Kim et al. 2014). Moreover, addition of TALE target sequences to the promoter of *Xa27* or *Xa10* resulted in resistance against additional *Xanthomonas* strains (Hummel et al. 2012; Zeng et al. 2015).

Screening strategies performed in *Nicotiana*, searching for cell death inducer genes by overexpression, were carried out in the past, but the designs were biased to paraquat or defense-related genes (Nasir et al. 2005; Coemans et al. 2008). Our approach using unbiased gene activation by PthA4^AT^ and dTALEs allowed the identification of genes involved in cell death that remained undiscovered in the former approaches. The success of our strategy validates the use of TALE proteins to scan genomes in search of additional cell death inducers and multiple other phenotypes.

## Materials and methods

### Bacterial growth conditions, plant material, and inoculation assays

Strains and plasmids used in this study are listed in Supplementary Tables S3 and S4, respectively. *Escherichia coli* and *Agrobacterium tumefaciens* (strain GV3101) were grown in LB medium at 37°C and 28°C, respectively. *Xanthomonas* were grown in peptone–yeast–malt (PYM) medium at 28°C. TNVA was propagated in *N. benthamiana* by mechanical inoculation as the source of virus. *N. benthamiana* plants were grown at 23°C and a photoperiod of 16-h light/8-h dark. Leaves of 6-week-old plants were inoculated by pressure infiltration with *Xcc*8004 or *Agrobacterium*. For *Xcc*8004 inoculation, bacterial suspensions were prepared in 10 mM MgCl_2_ [10^6^ colony-forming unit (CFU) ml^−1^], according to Chiesa et al. (2013). For *Agrobacterium* inoculation, overnight cultures were adjusted to OD_600_ = 0.4 in infiltration buffer (10 mM MES, 10 mM MgCl_2_, and 200 *μ*M acetosyringone in DMSO). Before infiltration, cultures were shaken for 2 h at room temperature. Overexpression constructs were co-infiltrated with a strain containing the *P19* gene, a suppressor of gene silencing. For TNVA inoculation 0.5 to 1 infective virions/*μ*l, crude leaf extract were used to rub-inoculate *N. benthamiana* plants at the two- to four-leaf stage (Garcia et al. 2023). Plants were monitored for up to 8 days at 22°C and 50% to 60% relative humidity.

### Microscopy

*Xcc8004* expressing PthA4^AT^: an cherry was monitored for 72 hpi using a confocal laser scanning microscope (Zeiss LSM 880; Carl Zeiss Microscopy GmBH Jena, Germany). The excitation of an cherry was accomplished with the 543 nm laser, and the emission was detected between 579 and 624 nm. Gain detection was set at 880. Chlorophyll was excited with the 405 nm laser line, and fluorescence was detected in the 649–704 nm range. Gain detection was set at 658. Simulated three-dimensional images and sections were generated by the ZEN 3.1 blue edition Free Viewer software. For nuclear co-localization, six *N. benthamiana* leaves were infiltrated with 10 mM Tris–KCl pH 7.4 buffer containing 10 *µ*g ml^−1^ DAPI (4′,6′-diamidino-2-phenylindole dihydrochloride) and incubated for 10 min. DAPI was excited with the 405-nm laser line, and the fluorescence was detected in the 438- to 479 nm range. Gain detections were set at 590. GFP was excited with the 488-nm laser line, and the fluorescence was detected in the 499 to 552 nm range. Chlorophyll was excited with the 405 nm laser line (15%) and fluorescence was detected in the 630 to 735 nm range. Simulated three-dimensional images and sections were generated by the ZEN 3.1 blue edition Free Viewer software. Nuclear localization for designer TALEs was monitored using a fluorescence lupe (MacroFluo MZ16F Leica Microsystems, USA). Two independent experiments were performed. In each experiment, three different leaves from three different plants per sample were used.

### Cloning strategies

dTals were constructed using the Golden TAL technology kit (Geißler et al. 2011). The selected RVDs were assembled to the destination vector pSKA2 adding the N- and C-terminal modules of the *Hax3* gene. To add repeats to the N-terminal end of the repeat region, the dTALEs require an N-terminal domain recognizing all four bases. Three amino acid changes (Q231R, W232G, S233A; Lamb et al. 2013) were incorporated into the Hax3 N-terminal domain by overlap extension PCR to build a generally applicable module for dTal assembly. Subsequently, pSKA2 constructions were transformed into *A. tumefaciens*. The pSKA2 vector fuses an N-terminal GFP epitope to the dTal. Coding sequences for dTal26-induced candidate targets and for the *PAT1* promoter were assembled using GoldenBraid (Vazquez-Vilar et al. 2017). Plasmids, constructs, and primers used are listed in Supplementary Tables S4 and S5.

### Conductivity assays

For ion leakage experiments, four 12-mm-diameter leaf discs were collected at 72 hpi in 5 ml of sterilized ultrapure water (Milli-Q) and incubated for 1 h in an orbital incubator (Stuart SI50, LabMakelaar Benelux B.V., The Netherlands) at 130 rpm and 25°C. Conductivity was measured using an ion conductivity meter (Basic C30, Crison, Spain). Three biological replicates per sample were performed. Each replicate contains four discs from four different leaves coming from two different plants.

### Luciferase activity measurements

The Dual-Glo Luciferase Assay System kit (Promega, USA) was used in this analysis. Firefly luciferase (FLuc) activity was measured using a GloMax Multi Detection System (Promega, USA), setting 10 s for measurement. After measuring, 40 *µ*l of Stop&Glo reagent was added per sample to quantify Renilla luciferase (RLuc) activity. FLuc/RLuc ratios were calculated as the mean value of three biological replicates. Each replicate consists of one 8-mm-diameter disc per infiltrated leaf collected at 36 hpi. Relative transcriptional activity was expressed as the FLuc/RLuc ratios normalized to the FLuc/RLuc ratios of the reference reporter Pnos:Luc (Supplementary Table S4).

### RNA purification

Total RNA was extracted using E.Z.N.A Plant RNA kit following RNase-free DNase (Omega Bio-Tek, USA) treatment. Three replicates per biological condition were performed. Each replicate contains leaf samples (100 mg) from three independent inoculated *N. benthamiana* plants randomly harvested at 36 hpi and pooled.

### RT-qPCR

cDNA was synthesized from 1 *μ*g of DNase-treated total RNA using Maxima First Strand cDNA Synthesis (Thermo Fisher Scientific, USA). RT-qPCRs were carried out using 1 *µ*g cDNA, 0.4 *µ*M primers, and 1× Pyrotaq EvaGreen qPCR Master Mix (Cultek Molecular Bioline, Spain). Three replicates per sample were performed. Relative transcript abundance was normalized against the reference F-Box gene using the ΔΔCt method (Livak and Schmittgen 2001). Primers are shown in Supplementary Table S5.

### RNA-Seq analysis and prediction of effector targets

Forty million 100 (for dTal26) or 150 (for dTal50 and dTal55) nucleotide paired-end reads per sample were obtained. Raw data were filtered using FastQC and Cutadapt v.2.10. Sequences were then mapped to the *N. benthamiana* genome (https://www.nbenth.com/) using HISAT2. Differential expression was estimated using DESEQ2 using RPKM (reads per kilobase per million reads) for normalization. PrediTALE (Erkes et al. 2019) was used for prediction of effector targets, considering regions spanning from 400 nt upstream to 200 nt downstream of the transcription start site (TSS). Promoter sequences were retrieved from the *N. benthamiana* (LAB330, version 3.01, https://www.nbenth.com/) reference genome.

### Accession numbers

Sequence data from this article can be found in GEO Omnibus database under the following accession numbers: GSE221293 and GSE221441. NEC3 accession number is NBlab07G09180; PAT1 accession number is NBlab18G05420. Sequences are available for downloading at www.nbenth.com.

## Supplementary Material

kiae230_Supplementary_Data

## Data Availability

RNA-Seq data have been deposited in GEO Omnibus database under the following accession numbers: GSE221293 and GSE221441.

## Supplementary data

The following materials are available in the online version of this article.

**Supplementary Figure S1.** Subcellular localization of PthA4^AT^ in *Nicotiana benthamiana*.

**Supplementary Figure S2.** Cell death response and tobacco necrosis virus A (TNVA) protection by dTal1 in *Nicotiana benthamiana*.

**Supplementary Figure S3.** Ambiguous repeat-variable diresidues (RVD) replacement to refine dTal1 specificity.

**Supplementary Figure S4.** Repeat-variable diresidues (RVDs) replacement over dTal26.

**Supplementary Figure S5.** Two rounds of repeat-variable diresidue (RVDs) extension at the N-terminal end of dTal27.

**Supplementary Figure S6.** Protection induced by dTal26 on *Nicotiana benthamiana* leaves rub-inoculated with tobacco necrosis virus A (TNVA).

**Supplementary Figure S7.** GFP signals in the nucleus of *Nicotiana benthamiana* leaves expressing dTal26 and photographed at 36 h post-inoculation (hpi).

**Supplementary Figure S8.** Promoter sequences of genes selected for overexpression in *Nicotiana benthamiana*.

**Supplementary Figure S9.** Repeat-variable diresidue (RVDs) extension on dTal27 C-terminal end.

**Supplementary Figure S10.** Strategy for refinement of dTALE target specificity used in this study.

**Supplementary Table S1.** dTal26-activated genes.

**Supplementary Table S2.** dTal-activated genes for dTal26, dTal50, and dTal55.

**Supplementary Table S3.** Bacterial strains and virus used in this study.

**Supplementary Table S4.** Plasmids used in this study.

**Supplementary Table S5.** List of primers used in this study.

## Dive Curated Terms

The following phenotypic, genotypic, and functional terms are of significance to the work described in this paper:

PYM Gramene: AT1G11400
PYM Araport: AT1G11400

PAT1 Gramene: AT3G56920
PAT1 Araport: AT3G56920

Chlorophyll CHEBI: CHEBI:28966
