# Supplementary material for: Designer TALEs enable discovery of cell death-inducer genes
Source: Plant Physiol. 2024 May 9;195(4):2985–96. doi: 10.1093/plphys/kiae230 (PMC11288752; doi:10.1093/plphys/kiae230)
Supplement: kiae230_Supplementary_Data [file kiae230_supplementary_data.zip › PP2023RA01410DR1_Supplemental_Material.pdf]

Designer transcription activator-like effectors enable discovery of cell death-inducer genes

Roxana A. Roeschlin, Sepideh M. Azad, René P. Grove, Ana Chuan, Lucila García, Regina Niñoles, Facundo Uviedo, Liara Villalobos-Piña, Maria E. Massimino, María R. Marano, Jens Boch and José Gadea

SUPPLEMENTARY DATA

Supplementary Figure S1 Subcellular localization of PthA4^{AT} in *Nicotiana benthamiana*. Confocal laser-scanning microscopy images of *N. benthamiana* leaves infiltrated with *Xcc*8004 expressing PthA4^{AT}:Cherry (magenta) and stained with DAPI (cyan). Arrows: nucleus. Green: Chloroplast. Scale bar: 20μm.

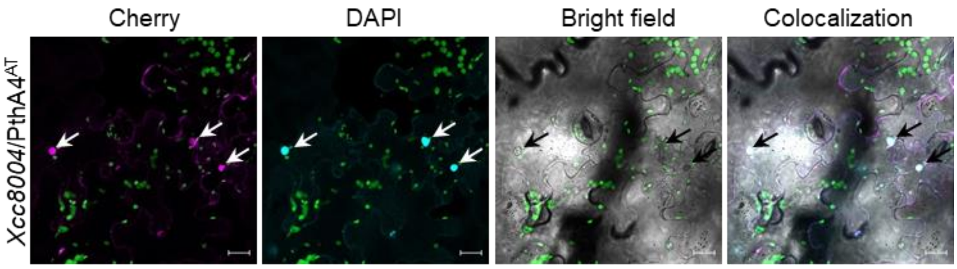

Supplementary Figure S2 Cell death response and tobacco necrosis virus A (TNVA) protection by dTal1 in *Nicotiana benthamiana*. A, Cell death response on leaves agroinfiltrated with PthA4^{AT} and dTal1. Photos were taken at 60 and 72 hours post inoculation (hpi). Control: infiltration medium. B, Necrotic lesions observed at 3 days post inoculation (dpi) on leaves rub inoculated with TNVA. *N. benthamiana* leaves were half-leaf agroinfiltrated with dTal1 and 4 days post-treatment inoculated with TNVA. Images were digitally extracted for comparison. Upper panel: Local protection of dTal1 was measured as the number of lesions per cm^2 in leaves at 3 dpi using ImageJ software v1.4. Values are expressed as means ± standard deviation of three independent biological replicates. *, significant after Tukey's test (p-value < 0.05). Lower panel: Systemic protection of dTal1 observed on distal leaves at 12 dpi. Control: *Agrobacterium* transformed with empty vector. Dashed lines indicate infiltrated area. Scale bar: 20 mm.

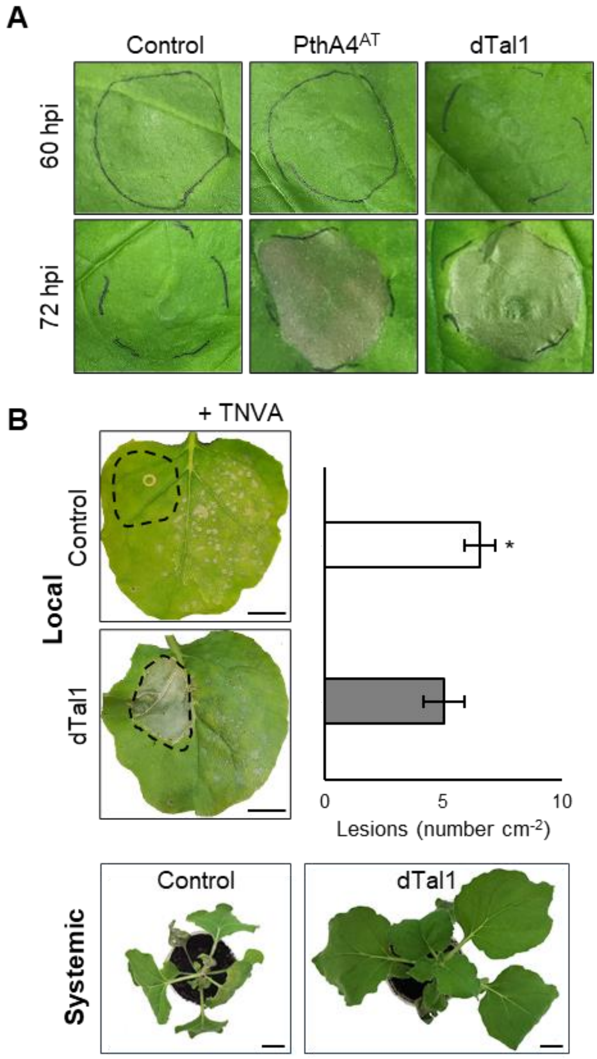

Supplementary Figure S3 Ambiguous repeat-variable diresidues (RVD) replacement to refine dTal1 specificity. A, RVDs of the different dTALEs. RVDs of dTal1 (PthA4^{AT}) are shown on yellow background. Red letters indicate RVD substitutions. B, Cell death response on *N. benthamiana* leaves infiltrated via *Agrobacterium*. Photos were taken at 60 and 72 h post inoculation (hpi). dTal10 triggered cell death covering the infiltrated area at 60 hpi, sooner than dTal1. dTal12 and dTal14 develop cell death at 72 hpi, suggesting that gene(s) triggering cell death could contain T and T/C on second and fourth position, respectively. CD: cell death. C, Cell damage evaluated by conductivity measurements at 72 hpi. Values are the mean ± standard deviation of three independent biological replicates. *, significant compared to control (one-way ANOVA, Tukey's test, p-value < 0.01). Control: infiltration medium.

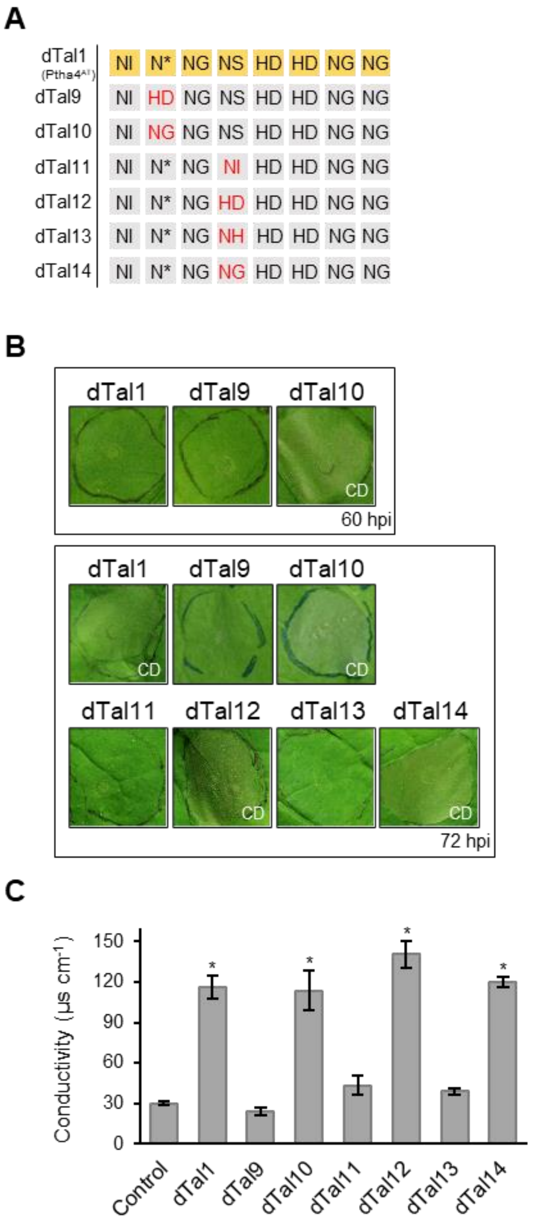

Supplementary Figure S4 Repeat-variable diresidues (RVDs) replacement over dTal26. A, Six new dTALEs (dTal27 to dTal32) were engineered substituting RVDs in position two (N*) and four (NS) in dTal26 by RVDs with better specificity for individual bases. RVDs of dTal1 (PthA4^{AT}) are shown on yellow background. Red letters indicate RVD substitutions and extensions. B, Cell death response at 60 hours post inoculation (hpi) for dTal27, dTal30 and dTal32 (magenta circles: cell death) confirming the requirement for T in position 2 and for T/C at position 4. White circles: no cell death; dashed lines: infiltrated area. Scale bar: 20mm.

A

dTal1 (Ptha4^{AT})	NI	N*	NG	NS	HD	HD	NG	NG		
dTal26	NI	N*	NG	NS	HD	HD	NG	NG	NH	NG
dTal27	NI	NG	NG	NS	HD	HD	NG	NG	NH	NG
dTal28	NI	HD	NG	NS	HD	HD	NG	NG	NH	NG
dTal29	NI	N*	NG	NI	HD	HD	NG	NG	NH	NG
dTal30	NI	N*	NG	HD	HD	HD	NG	NG	NH	NG
dTal31	NI	N*	NG	NH	HD	HD	NG	NG	NH	NG
dTal32	NI	N*	NG	NG	HD	HD	NG	NG	NH	NG

B

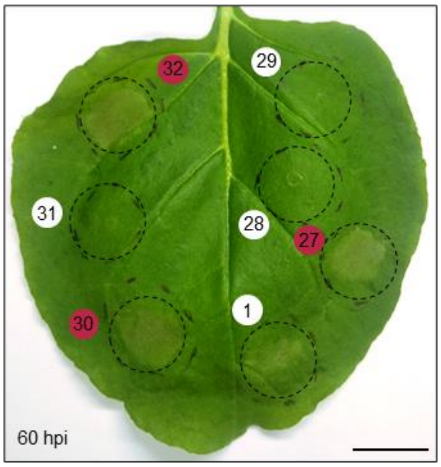

Supplementary Figure S5 Two rounds of repeat-variable diresidue (RVDs) extension at the N-terminal end of dTal27. A, RVDs of the different dTALEs. RVDs of dTal1 (PthA4^{AT}) are shown on yellow background. Red letters indicate RVD substitutions and extensions. B, Cell death response on *N. benthamiana* leaves infiltrated via *Agrobacterium* and photographed at 72 h post inoculation (hpi). Magenta circles: cell death; white circles: no cell death; dashed lines: infiltrated area; C: infiltration medium. Scale bar: 20mm.

A

dTal1 (Ptha4^{AT})				NI	N*	NG	NS	HD	HD	NG	NG		
dTal27				NI	NG	NG	NS	HD	HD	NG	NG	NH	NG
dTal57		NI	NG	NI	NG	NG	NS	HD	HD	NG	NG	NH	NG
dTal58		HD	NG	NI	NG	NG	NS	HD	HD	NG	NG	NH	NG
dTal59		NH	NG	NI	NG	NG	NS	HD	HD	NG	NG	NH	NG
dTal60		NG	NG	NI	NG	NG	NS	HD	HD	NG	NG	NH	NG
dTal61	NI	NG	NG	NI	NG	NG	NS	HD	HD	NG	NG	NH	NG
dTal62	HD	NG	NG	NI	NG	NG	NS	HD	HD	NG	NG	NH	NG
dTal63	NH	NG	NG	NI	NG	NG	NS	HD	HD	NG	NG	NH	NG
dTal64	NG	NG	NG	NI	NG	NG	NS	HD	HD	NG	NG	NH	NG

B

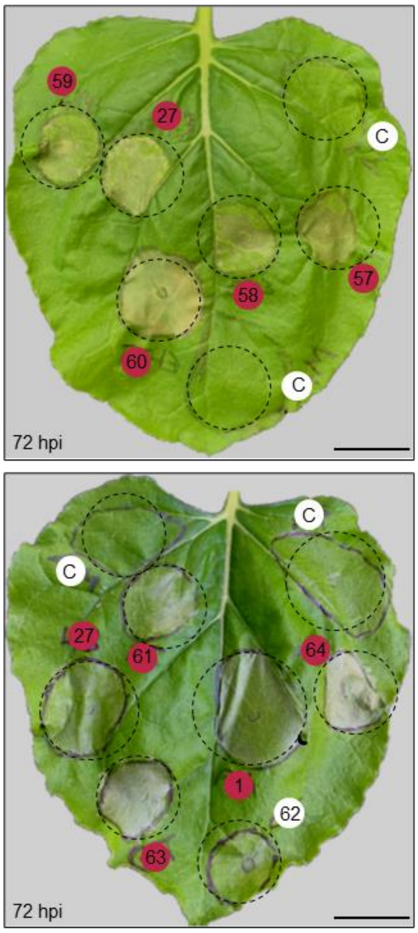

Supplementary Figure S6 Protection induced by dTal26 on *Nicotiana benthamiana* leaves rub inoculated with tobacco necrosis virus A (TNVA). A, Necrotic lesions observed at 3 days post inoculation (dpi) on leaves rub inoculated with TNVA. *N. benthamiana* leaves were half-leaf agroinfiltrated with dTal26 and 4 days post-treatment inoculated with TNVA. Dashed circles: infiltrated area. Local protection of dTal26 was measured as the number of lesions per cm^2 in leaves at 3 dpi using ImageJ software v1.4. Values are expressed as means ± standard deviation of three independent biological replicates. *, significant after Tukey's test (p-value < 0.05). B, Systemic protection of dTal26 observed on distal leaves at 12 dpi. Images were digitally extracted for comparison. Control: *Agrobacterium* transformed with empty vector. Scale bar: 20 mm.

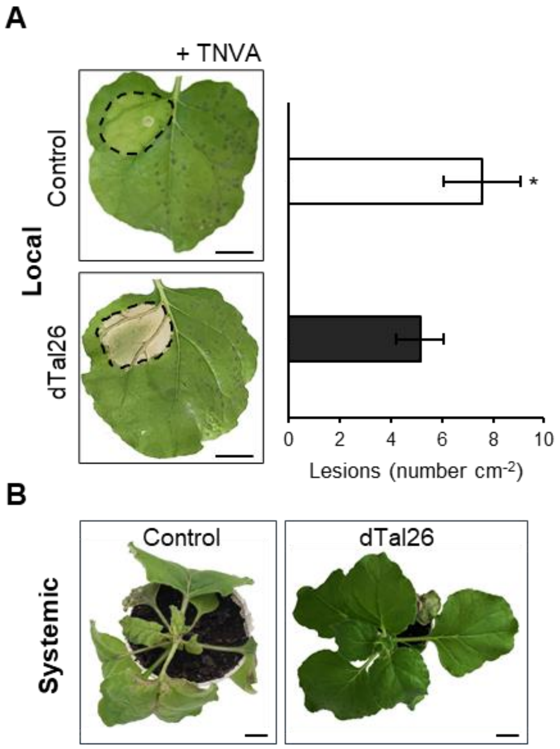

Supplementary Figure S7 GFP signals in the nucleus of *Nicotiana benthamiana* leaves expressing dTal26 and photographed at 36 hours post inoculation (hpi). GFP: cyan; chloroplast: magenta; white arrows: nucleus. Scale bar: 20µm.

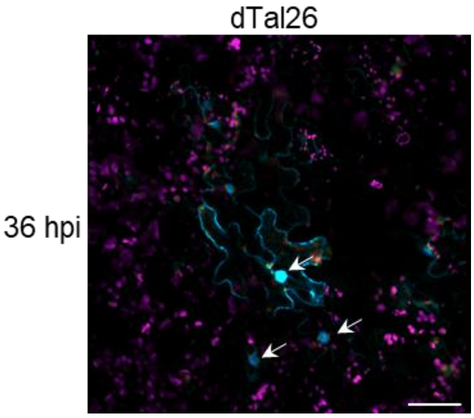

Supplementary Figure S8 Promoter sequences of genes selected for overexpression in *Nicotiana benthamiana.* dTal26 effector-binding-site according to PrediTALE (p-value < 10^{-5}) is underlined. 5´ end of the TALE predicted binding site is highlighted.

>NBlab04G00720_RLP21
GTCATAAAATTGAAATCATGGCTCCGCTTCTACATGAACTCACTTGTTTGCAAAAAAAAAGA
TTCTATTAGACTGTTAGTGATATAACTCATAAAGGGGAGCTTTGGTGTAACCGGTAAAGTTA
CTATTATGTGACTAGAATGTCACAAGTTCGAGTAGTGAAAATAGCCACTTGTTAGGAGGTCA
CGGGTTCAAGCCGTGGAAACAATCACTTGTCCGGAAGTCACGGGTTTTAAGTCGTGGAAA
CAGTGATCACATGCCGAAATGCCCCCGCACATAACGAGAACTTAGTGTACTCAGACAGCTT
TTTTTGTTAGCGATATAACTCATGCGTGCAACTAACTAACTAACTTTTTATATATATATATATA
TATATATATATATATATAGGGATAATGATAATTGATTTTGTAAGAACATTCTGTTATTTGCTGG
GAAAAAAAGGGAAGTATCTTTTTCTTTTATTCACTTGTCTGTGTTGTGTTGCTAAGTAAAAGT
TGTTGTTGTTCTTCTTCGCTTGAGCATCATCTTTGCTCTCCTAGTGAAGCTTTTGCTTTGCTC
CAGTTCAAGCAACCCTTTGAAATATACAACGAGTCTAGT
>NBlab04G26950_MYB5
AATAGAAAATGTATGCCTACAATCATGAATATTATGAAATTTTGAGAAACAAAATAATCTTAG
TTTGGTCTCTATCCTGGCCACAACGTGATTACCTCTCTATAAAAAAATACAAGTGTACGACA
TGATAACGAATCTAAGTTGTAGATCCACGAAAAACATGCACAAGGGGAAATAAAATATTTGC
ATAACAACAAACCAATCAATTCATTCCCCAATAAATAAGGAACAACTAGATCTGACTGTCAT
TTACTTTAATTATTTTTCTCTCAGACATTTATTATTATTATTATTATTATTATTATTATTATA
TAGTATTAATTATTGCCTTGGAAATTTCAACCTCGTGGCTTCAAAAGAGCACCACCTTTCCT
TTAAATCCTATCTTCACTTCACTCTTCACTTCTTCTTAGTTCTCTCTGTTGAGCTCTGAAAAT
TTACTAAAATAAGAAGGGAAAAAAAGGGAAAAAGAAATTAAGAAATGGTTAGAAAACCATGT
TGTGTAAAAGAGGGATTGAGAAAAGGTCCATGGTCATCTAAAGAAGATTTATTACTTACTAA
TTATATCAAGGAAAATGGTGAAGGACAATGGAGATC
>NBlab12G27340_NAC79
AGTTGGAACCACCATGAAAGCAACATTTGGAGCTTTCAAACTATTCACACCAGAGAATGAA
GTTCAAAAAATTAAATGAATATGACTATAGTTAAAATAAAATGACAAAAGCAGTTGAAAATGT
TACATGCATGGTTTTAGGGATGAGTTGTATTTAGTCAAATTATAAAAAAAAGGTGGATAGAT
CTTTTATATAATGGTTAGGAAAAGAGTTAAAAGAGAAGGCCTAAACAACTAATCAGTGTTGG
GTTTGGTGGAGTGGTAAATACTTCTTTATTTTTAATTAGAGATTTCGAATTCGAATTTTAAAT
ATAAAATTACCTTTGCCTACCCAGTATGATACTTATTGGTAAGAATCTTGATTTAGTGGAACC
TTAATACAAATATCGAACCGTTGATGGGAAACTAAAAAGAAGGCCTAGACATCTTCCACCCT
CCCCCACCCACCCAAAACCTCTATTTATTGCCTTGTATTCCTCATACTTCTTTTTTTCATTCT
TTCAGTTTAGTTCCATGGCTTCTCTTTACCTTTTTTCTCCAACTTCCCCATTTCTTCCTACTTT
TCTTGATTAAGTATTCTTTCTCATGGTCTTTTGAGCT
>NBlab07G12620_NAC100
CTAAAATAAAATGACAAAAGCAGTTGAAAATGTTACATGCATGGTTTTAGGGATAAGTTGTA
TTTGGTCAAACAATAAACAAAAAGTGGATATATTTTTTGTATCACGGTTAGGAAAAGAGTTAA
AGAGAAGGCCTAAACAACTGATCAAAGTTGTGATGGAGTGATAAATACTTATTCATTTTTAA
TTAGAAATCGCGAATTCGTACTATGAGTATAAAATCATCTTTGTCGGAAGTACTTTACCCTC
AATATCAAACTTACCGATGTGAATTATGATTTAGTCGAGTCCTAGTGCGGATAGAAAAAAAG
GCCCAGACACCTTCCTCCCCCCTCCCCCAACAAAATATCTATTTATTGCCTTGTATTCCTCA
TACTTCTTTTAATAAAAATTCTGTCAGTTTTAGTTCCATGGATTCTCTTTTATCTTTTTCTCCG
TCTTCTCCATTTCTTCCAACTTTTCTTGATTTAGTATTCTTTCTCACGGTCTTTTGAGCTGTTT
GGCCAAACCCCATTTATATTTCAGAGCCAGCTGGTGAGCGGTGGTCAATATAATCTTTATTA
GTGTGATTATAGGTGAAATAATTAATTCTTGATTACA

Supplementary Figure S9 Repeat-variable diresidue (RVDs) extension on dTal27 C-terminal end. A, Upper-panel, RVDs of engineered dTALEs. Eight dTALEs (dTal33-dTal40) were generated with two repeats added to the C-terminal end of the dTal27 repeat domain. RVDs of dTal1 ($PthA4^{AT}$) are shown on yellow background. Red letters indicate RVD substitutions and extensions. Given the low specificity of the last RVD in previous rounds of extension, the next target base (corresponding to the 11th RVD in the dTALE) was evaluated by placing the interrogated RVD in the penultimate position, followed by one of two randomly chosen RVDs. Thus, dTal33 (dTal27-NI-NI) and dTal34 (dTal27-NI-NG) evaluated the preference for A, dTal35 (dTal27-HD-NG) and dTal36 (dTale27-HD-NI) the preference for C, dTal37 (dTal27-NH-HD) and dTal38 (dTal27-NH-NG) for G and dTal39 (dTal27-NG-HD) and dTal40 (dTal27-NG-NG) for T in the penultimate (11th) position. Lower panel, cell death response on *N. benthamiana* leaves infiltrated via *Agrobacterium* and photographed at 60 hours post inoculation (hpi). dTal39 followed similar cell death induction dynamics than dTale26 and dTale27, showing advanced cell death lesions at 60 hpi. dTale38 and dTale40 developed a weaker cell death response at 60 hpi. Red circles: cell death. B, Upper-panel, RVDs of engineered dTALEs. An additional round of extension was designed to evaluate position 12, using dTal39 up to the penultimate position as the starting dTALE (dTal39-1), following the same scheme as above. A strong cell death following agroinfiltration into *N. benthamiana* was observed in the infiltrated area at 72 hpi for dTal46, dTal47, and dTal48 and a weaker response for dTal45. Images were digitally extracted for comparison. Magenta circles: cell death; white circles: no cell death; dashed lines: infiltrated area; C: infiltration medium. Scale bar: 20mm.

A

dTal1 (Ptha4^{AT})	NI	N*	NG	NS	HD	HD	NG	NG				
dTal27	NI	NG	NG	NS	HD	HD	NG	NG	NH	NG		
dTal33	NI	NG	NG	NS	HD	HD	NG	NG	NH	NG	NI	NI
dTal34	NI	NG	NG	NS	HD	HD	NG	NG	NH	NG	NI	NG
dTal35	NI	NG	NG	NS	HD	HD	NG	NG	NH	NG	HD	NG
dTal36	NI	NG	NG	NS	HD	HD	NG	NG	NH	NG	HD	NI
dTal37	NI	NG	NG	NS	HD	HD	NG	NG	NH	NG	NH	HD
dTal38	NI	NG	NG	NS	HD	HD	NG	NG	NH	NG	NH	NG
dTal39	NI	NG	NG	NS	HD	HD	NG	NG	NH	NG	NG	HD
dTal40	NI	NG	NG	NS	HD	HD	NG	NG	NH	NG	NG	NG

B

dTal1 (Ptha4^{AT})	NI	N*	NG	NS	HD	HD	NG	NG					
dTal27	NI	NG	NG	NS	HD	HD	NG	NG	NH	NG			
dTal39	NI	NG	NG	NS	HD	HD	NG	NG	NH	NG	NG	HD	
dTal41	NI	NG	NG	NS	HD	HD	NG	NG	NH	NG	NG	HD	NG
dTal42	NI	NG	NG	NS	HD	HD	NG	NG	NH	NG	NG	HD	NI
dTal43	NI	NG	NG	NS	HD	HD	NG	NG	NH	NG	NG	NG	NG
dTal44	NI	NG	NG	NS	HD	HD	NG	NG	NH	NG	NG	NG	NI
dTal45	NI	NG	NG	NS	HD	HD	NG	NG	NH	NG	NG	NI	NG
dTal46	NI	NG	NG	NS	HD	HD	NG	NG	NH	NG	NG	NI	NI
dTal47	NI	NG	NG	NS	HD	HD	NG	NG	NH	NG	NG	NH	NG
dTal48	NI	NG	NG	NS	HD	HD	NG	NG	NH	NG	NG	NH	NI

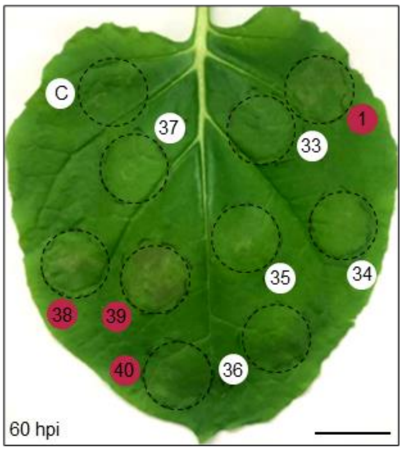

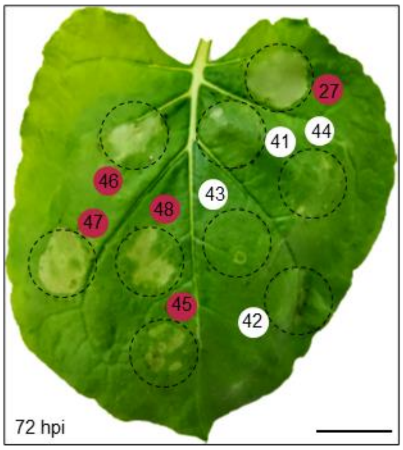

Supplementary Figure S10 Strategy for refinement of dTALE target specificity used in this study. A, Schematic representation for DNA-target specificity of TALEs. (Top) TALE domains include an N-terminal region for type III secretion (T3SS) and non-canonical repeats (NCR), a central repeat region, and a C-terminal domain with a transcription factor binding (TFB), nuclear localization signals (NLS) and an acidic activation domain (AAD). The amino acid sequence of a repeat and the repeat-variable diresidue (RVD) in position 12 and 13 are shaded in red. (Bottom) RVDs of artificial TALEs and target sequence. B, Designer TALEs to reduce potential target sites of PthA4^{AT}.

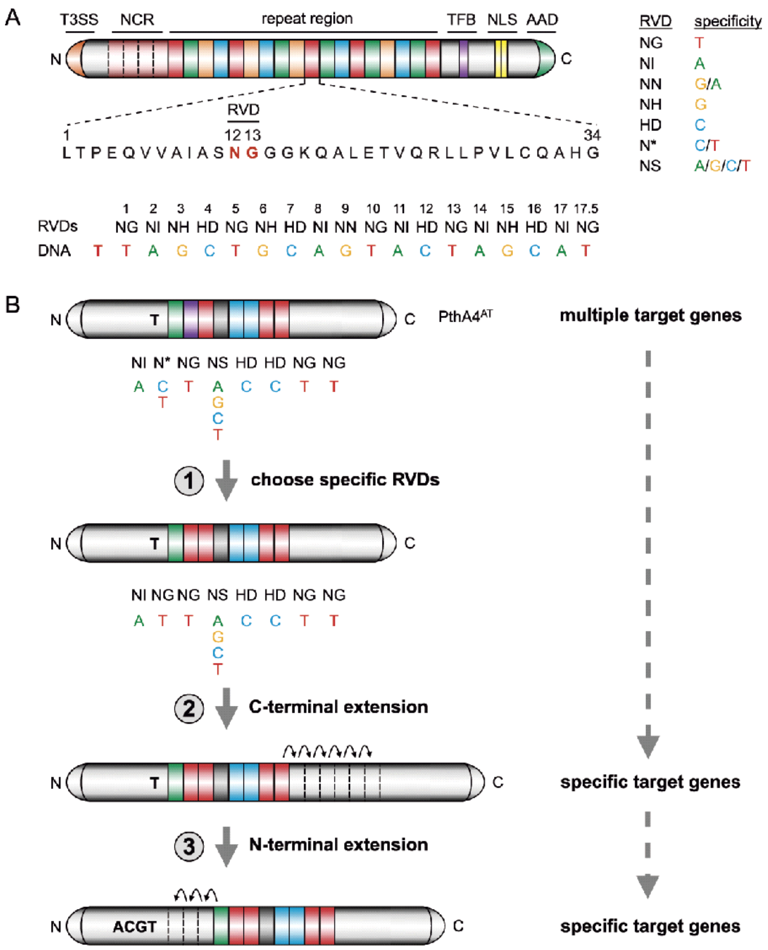

Supplementary Table S1. dTal26-activated genes. Criteria used for selection: average expression after infiltration with dTale9 lower than 2 RPKM, Log2FoldChange dTal26/dTal9 > 4, PrediTALE p-value < 10^{-5}. (Excel File).

Supplementary Table S2 dTal-activated genes for dTal26, dTal50 and dTal55 according to the following criteria: Log_2FoldChange > 1, adjusted p-value < 0.05 for expression, p-value < 10^{-5} for PrediTALE prediction. (Excel File).

Supplementary Table S3. Bacterial strains and virus used in this study.

Name	Relevant characteristics	Reference
Xanthomonas campestris pv. campestris (Xcc)		
Xcc8004	Wild type; Rif^R	(Daniels et al., 1984)
Xcc/PthA4^{AT}	*Xcc*8004 carries pBBR-*pthA4^{AT}*; Rif^R, Km^R	This study
Xcc/mutNLS^{AT}	*Xcc*8004 carries pBBR-*mutNLS^{AT}*, Rif^R, Km^R	This study
Xcc/PthA4^{AT}:Cherry	*Xcc*8004 carries pBRR-*pthA4^{AT}*:Cherry, Km^R	This study
Agrobacterium tumefasciens		
GV3101	C58C1	(Van Larebeke et al., 1974)
PthA4^{AT}	GV3101 carries pCHF3 containing 35S fused with *pthA4^{AT}* gene, Spc^R	(Roeschlin et al., 2019)
mutNLS^{AT}	GV3101 carries pCHF3 containing *pthA4*^{AT} gene with point mutations on NLS domain, Spc^R	(Roeschlin et al., 2019)
dTal	GV3101 carries pSKA2-dTALEs, Spc^R	This study
35S::PAT1	GV3101 carries 35S:*PAT1*:Tnos, Spc^R	This study
pPAT1	GV3101 carries pPAT1:Luc:Tnos-SF-35S:Renilla:Tnos-35S:P19:Tnos, Spc^R	This study
Pnos:Luc	GV3101 carries Pnos:Luciferase:Tnos-SF-35S:Renilla:Tnos-35S:P19:Tnos reference reporter, Spc^R	This study
35S::NEC3	GV3101 carries 35S:*NEC3*:Tnos, Spc^R	This study
Escherichia coli		
E. coli DH5α	*hsdR recA lacZYA* ϕ80 *lac*ZΔM15	ThermoFisher Scientific, Waltham, MA, USA
E. coli ccdB	*ccdB Survival™ 2 T1^R*	ThermoFisher Scientific, Waltham, MA, USA
Tobacco necrosis virus		
TNVA	Tobacco necrosis virus A	(Garcia et al., 2023)

Rif^R, rifampicin resistance; Km^R, kanamycin resistance; Spc^R, spectinomycin resistance.

Daniels MJ, Barber CE, Turner PC, Cleary WG, Sawczyc MK (1984) Isolation of mutants of Xanthomonas campestris pv. campestris showing altered pathogenicity. J Gen Microbiol 2447–2455

Garcia L, Gerhardt N, Martin AP, Martínez MF, Alemano S, Marano MR (2023) Tobacco necrosis virus A overcomes local cell death response in Nicotiana tabacum. Plant Pathol **72**: 154–169

Van Larebeke N, Engler G, Holsters M, Van Den Elsacker S, Zaenen I, Schilperoort RA, Schell J (1974) Large plasmid in Agrobacterium tumefaciens essential for crown gall-inducing ability. Nature **252**: 169–170

Roeschlin RA, Uviedo F, García L, Molina MC, Favaro MA, Chiesa MA, Tasselli S, Franco-Zorrilla JM, Forment J, Gadea J, et al (2019) PthA4AT, a 7.5-repeats transcription activator-like (TAL) effector from *Xanthomonas citri* ssp. *citri*, triggers citrus canker resistance. Mol Plant Pathol **20**: 1394–1407

Supplementary Table S4. Plasmids used in this study.

Plasmids	***Relevant genotype***	***Referenc e***
	Xcc8004 transformation	
pBBR	pBBR1-MCS2 derivative, mob-site, LacZα+; Km^R	(Kovach et al., 1995)
pBBR-pthA4^{AT}	pBBR vector containing *pthA4* gene of *X. citri* A^T; Km^R	(Roeschlin et al., 2019)
pBBR-mutNLS^{AT}	pBBR containing pthA4^{AT} gene with point mutations on NLS domain, Km^R	(Roeschlin et al., 2019)
pBBR-cherry	pBBR vector containing Cherry fluorescent epitope; Km^R	This study
pBBR-pthA4^{AT}:cherry	pBBR vector containing *pthA4* gene of *X. citri* A^T with C-terminal fused to Cherry fluorescent epitope; Km^R	This study
	dTale construction	
RVDs	RVDs (NI, NG, NH, HD, NS, NN o N*) containing *Bpi*I and *Bsa*I cloning restriction enzymes sites; Ap^R	(Geißler et al., 2011)
N-terminal (NTH3)	N-terminal from Hax3 (*X. armoraciae*) fused to GFP with *Bsa*I restriction site; Ap^R	(Geißler et al., 2011)
NTH3-m	N-terminal from Hax3 (*X. armoraciae*) modified for N-terminal extensions fused to GFP with *Bsa*I restriction site; Ap^R	This study
C-terminal (CTM WS)	C-terminal from Hax3 (*X. armoraciae*) with *Bsa*I restriction site; Ap^R	(Geißler et al., 2011)
AV (LR, LA, AR, AB, BR, BC, CR)	Assembly vector with *Bsa*I "L left" extreme binding NTH3 vector, *Bpi*I internal site with *lacZ* gene and *Bsa*I "R right" extreme binding CTM WS vector; (*Bsa*I – repeat border – *Bpi*I l*acZ Bpi*I – repeat border - *Bsa*I); Km^R	(Geißler et al., 2011)
pSKA2	pSKA2 vector for dTal expression in *N. benthamiana*; pVM_BGW backbone, LB- 35S pro, NTM_eGFP, *Bsa*I *ccdB Bsa*I – RB; Spc^R	(Geißler et al., 2011)
	pSKA2 expressing dTales	
dTal1	NI N* NG NS HD HD NG NG	This study
dTal2	HD N* NG NS HD HD NG NG	This study
dTal3	NI NN NG NS HD HD NG NG	This study
dTal4	NI N* NI NS HD HD NG NG	This study
dTal5	NI N* NG NS NN HD NG NG	This study
dTal6	NI N* NG NS HD NN NG NG	This study
dTal7	NI N* NG NS HD HD NN NG	This study
dTal8	NI N* NG NS HD HD NG NN	This study
dTal9	NI HD NG NS HD HD NG NG	This study
dTal10	NI NG NG NS HD HD NG NG	This study
dTal11	NI N* NG NI HD HD NG NG	This study
dTal12	NI N* NG HD HD HD NG NG	This study
dTal13	NI N* NG NH HD HD NG NG	This study

dTal14	NI N* NG NG HD HD NG NG	This study
dTal15	NI N* NG NS HD HD NG NG NI	This study
dTal16	NI N* NG NS HD HD NG NG HD	This study
dTal17	NI N* NG NS HD HD NG NG NN	This study
dTal18	NI N* NG NS HD HD NG NG NG	This study
dTal19	NI N* NG NS HD HD NG NG HD NI	This study
dTal20	NI N* NG NS HD HD NG NG HD HD	This study
dTal21	NI N* NG NS HD HD NG NG HD NN	This study
dTal22	NI N* NG NS HD HD NG NG HD NG	This study
dTal23	NI N* NG NS HD HD NG NG NH NI	This study
dTal24	NI N* NG NS HD HD NG NG NH HD	This study
dTal25	NI N* NG NS HD HD NG NG NH NN	This study
dTal26	NI N* NG NS HD HD NG NG NH NG	This study
dTal27	NI NG NG NS HD HD NG NG NH NG	This study
dTal28	NI HD NG NS HD HD NG NG NH NG	This study
dTal29	NI N* NG NI HD HD NG NG NH NG	This study
dTal30	NI N* NG HD HD HD NG NG NH NG	This study
dTal31	NI N* NG NH HD HD NG NG NH NG	This study
dTal32	NI N* NG NG HD HD NG NG NH NG	This study
dTal33	NI NG NG NS HD HD NG NG NH NG NI NI	This study
dTal34	NI NG NG NS HD HD NG NG NH NG NI NG	This study
dTal35	NI NG NG NS HD HD NG NG NH NG HD NG	This study
dTal36	NI NG NG NS HD HD NG NG NH NG HD NI	This study
dTal37	NI NG NG NS HD HD NG NG NH NG NH HD	This study
dTal38	NI NG NG NS HD HD NG NG NH NG NH NG	This study
dTal39	NI NG NG NS HD HD NG NG NH NG NG HD	This study
dTal40	NI NG NG NS HD HD NG NG NH NG NG NG	This study
dTal41	NI NG NG NS HD HD NG NG NH NG NG HD NG	This study
dTal42	NI NG NG NS HD HD NG NG NH NG NG HD NI	This study
dTal43	NI NG NG NS HD HD NG NG NH NG NG NG NG	This study
dTal44	NI NG NG NS HD HD NG NG NH NG NG NG NI	This study
dTal45	NI NG NG NS HD HD NG NG NH NG NG NI NG	This study
dTal46	NI NG NG NS HD HD NG NG NH NG NG NI NI	This study
dTal47	NI NG NG NS HD HD NG NG NH NG NG NH NG	This study
dTal48	NI NG NG NS HD HD NG NG NH NG NG NH NI	This study
dTal49	NI NG NG NS HD HD NG NG NH NG NG NI NI NG	This study
dTal50	NI NG NG NS HD HD NG NG NH NG NG NI NG NG	This study
dTal51	NI NG NG NS HD HD NG NG NH NG NG NI NH NG	This study
dTal52	NI NG NG NS HD HD NG NG NH NG NG NI HD NG	This study
dTal53	NI NG NG NS HD HD NG NG NH NG NG NH NI NG	This study
dTal54	NI NG NG NS HD HD NG NG NH NG NG NH NG NG	This study
dTal55	NI NG NG NS HD HD NG NG NH NG NG NH NH NG	This study
dTal56	NI NG NG NS HD HD NG NG NH NG NG NH HD NG	This study
dTal57	NI NG NI NG NG NS HD HD NG NG NH NG	This study
dTal58	HD NG NI NG NG NS HD HD NG NG NH NG	This study

dTal59	NH NG NI NG NG NS HD HD NG NG NH NG	This study
dTal60	NG NG NI NG NG NS HD HD NG NG NH NG	This study
dTal61	NI NG NG NI NG NG NS HD HD NG NG NH NG	This study
dTal62	HD NG NG NI NG NG NS HD HD NG NG NH NG	This study
dTal63	NH NG NG NI NG NG NS HD HD NG NG NH NG	This study
dTal64	NG NG NG NI NG NG NS HD HD NG NG NH NG	This study
dTalG1.1	NI NG NG NH NI HD NI NH NI NH NI HD NI HD NG NG NG NI NI NG	This study
dTalG1.2	NI NG NG NG NI NG NG HD HD NI HD NI HD NG NH NI NG	This study
dTalG1.3	NI NG NH NI NG NG NI NG HD NI NG HD NG NG NG NH NI NH NI NI NH NI HD NG	This study
dTalG1.4	HD NG NG NG NH NI NH NI NI NH NI HD NG NH HD NI NG NI NG NI NG	This study
dTalG2.1	NN NG HD NI NI NI NI NN HD NI NI HD NG NG HD NG NG NG	This study
dTalG2.2	NI NG NI NG NI NI NG NN NI NG HD HD NI NI NN NI NN NG	This study
dTalG2.3	NN NN NG HD NI NN HD NG NG HD NI HD NI NG NG NI HD HD	This study
dTalG2.4	HD NN HD NI NG NG NI HD NI NI NN NG NG NG NG NN NG HD	This study
dTalG2.5	HD HD NI NI NN NI NN NG NG NG NG NN NG NN NI NI NG NG	This study
dTalG2.6	NN NG NG HD NG HD HD HD HD NG NI NG NI NG NI NI NG NN	This study
	Golden Bride cloning system	
35S	pUPD vector containing CaMV 35S promoter. (Golden Braid ID: GB0030); Ap^R	gbcloning.upv.es
nos Terminator	pUPD vector containing nos Terminator. (Golden Braid ID: GB0037); Ap^R	gbcloning.upv.es
CDS Luciferase	pUPD vector containing CDS Luciferase. (Golden Braid ID: GB0096); Ap^R	gbcloning.upv.es
pUPD2	pSB1C3 cloning vector; Cm^R	gbcloning.upv.es
pEGB SF	pDGB3_alpha2 vector containing the transcriptional units. Twister plasmid to swap inserts from an alpha1 or alpha1R vector to any omega level vector (Golden Braid ID: GB1236); Km^R	gbcloning.upv.es
SF-35S:Renilla:Tnos-35S:P19:Tnos	pDGB1_alpha2 vector for the expression of the Renilla Luciferase with the silencing suppressor P19 (Golden Braid ID: GB0160); Km^R	gbcloning.upv.es
Pnos:Luc	pDGB1_omega1 vector containing Pnos:Luciferase:Tnos-SF-35S:Renilla:Tnos-35S:P19:Tnos for the expression of the Firefly Luciferase, the Renilla Luciferase and the P19 silencing suppressor. Reference reporter (Golden Braid ID: GB0166); Spc^R	gbcloning.upv.es
35S:PAT1:Tnos	pDGB3_omega1 destination vector with 35S promoter fused to PAT1 gene from *N. benthamiana* (NBlab18G05420) and the nos Terminator; Spc^R	This study
pPAT1:Luc:Tnos	pDGB3_omega1 destination vector with pPAT1:Luc:Tnos-SF-35S:Renilla:Tnos-35S:P19:Tnos. pPAT1; Spc^R	This study
35S:RLP21:Tnos	pDGB3_omega1 destination vector with 35S promoter fused to RLP21 gene from *N. benthamiana* (NBlab04G00720) and the nos Terminator; Spc^R	This study

35S:MYB5:Tnos	pDGB3_omega1 destination vector with 35S promoter fused to MYB5 gene from *N. benthamiana* (NBlab04G26950) and the nos Terminator; Spc^R	This study
35S:NAC79:Tnos	pDGB3_omega1 destination vector with 35S promoter fused to NAC79 gene from *N. benthamiana* (NBlab12G27340) and the nos Terminator; Spc^R	This study
35S:NAC100:Tnos	pDGB3_omega1 destination vector with 35S promoter fused to NAC100 gene from *N. benthamiana* (NBlab07G12620) and the nos Terminator; Spc^R	This study
35S:NEC3:Tnos	pDGB3_omega1 destination vector with 35S promoter fused to MYB5 gene from *N. benthamiana* (NBlab07G09180) and the nos Terminator; Spc^R	This study

Ap^R, ampicillin resistance; Cm^R, chloramphenicol resistance; Km^R, kanamycin resistance Spc^R, spectinomycin resistance.

Geißler R, Scholze H, Hahn S, Streubel J, Bonas U, Behrens SE, Boch J (2011) Transcriptional activators of human genes with programmable DNA-specificity. PLoS One **6**: e19509

Kovach ME, Elzer PH, Steven Hill D, Robertson GT, Farris MA, Roop RM, Peterson KM (1995) Four new derivatives of the broad-host-range cloning vector pBBR1MCS, carrying different antibiotic-resistance cassettes. Gene **166**: 175–176

Roeschlin RA, Uviedo F, García L, Molina MC, Favaro MA, Chiesa MA, Tasselli S, Franco-Zorrilla JM, Forment J, Gadea J, et al (2019) PthA4AT, a 7.5-repeats transcription activator-like (TAL) effector from *Xanthomonas citri* ssp. *citri*, triggers citrus canker resistance. Mol Plant Pathol **20**: 1394–1407

Supplementary Table S5. List of primers used in this study.

**Primer name**	**Sequence (5’□3’)**
**CDS amplification**	
PAT1 (fw)_1<br>PAT1 (rv)_1	GCGCCGTCTCGCTCGAATGGAAAACGAAGCAGCGGG<br>GCGCCGTCTCGCTCAAAGCCTAGACCAACCGGCTACTGG
PAT1 (fw)_2<br>PAT1 (rv)_2	GCGCCGTCTCGCTCGGGAGTGGAATTTTTTTAAAACAAATATCGTGC<br>GCGCCGTCTCGCTCACATTTATCAACGATAAGAGTAAAACAATATATA
RLP21 (fw)<br>RLP21 (rv)	GCGCCGTCTCGCTCGAATGCTTGAAGCGTTAGATCTC<br>GCGCCGTCTCGCTCAAAGCTTAAGGCCTTCTTCTCTGAGC
MYB5 (fw)<br>MYB5 (rv)	GCGCCGTCTCGCTCAAAGCTTATACCTTGGGCTCCTGCA<br>GCGCCGTCTCGCTCAAAGCTCACTGATCAGAGTCATTTACAG
NAC79 (fw)<br>NAC79 (rv)	GCGCCGTCTCGCTCGAATGGAAAATGATTCGGGACTTG<br>GCGCCGTCTCGCTCAAAGCTCAGTAACTCCAGAGGCAATC
NAC100 (fw)<br>NAC100 (rv)	GCGCCGTCTCGCTCGAATGGAAAATTATTTGGGACTTGTTAA<br>GCGCCGTCTCGCTCAAAGCTCAGTAACTCCAAAGGCAATC
NEC3 (fw)<br>NEC3 (rv)	GCGCCGTCTCGCTCGATGGCAGCAACGAAAATTG<br>GCGCCGTCTCGCTCAAAGCTCAGGGCCTTTCTTCCGGTT
**Promoter amplification**	
pPAT1 (fw)<br>pPAT1 (rv)	GCGCCGTCTCGCTCGGGAGTGGAATTTTTTTAAAACAAATATCGTGC<br>GCGCCGTCTCGCTCACATTTATCAACGATAAGAGTAAAACAATATATA
**qRT-PCR**	
PAT1 (fw)q<br>PAT1 (rv)q	GCAATCTCGCGACCTGATTT<br>TATTAGCAGCCACACCACCA
F-Box (fw)q<br>F-Box (rv)q	GGCACTCACAAACGTCTATTTC<br>ACCTGGGAGGCATCCTGCTTAT
NEC3 (fw)<br>NEC3 (rv)	GTTGTATGGACTATTGATAGAAAGGTGAAC<br>ATCATTTCACTTCATTTTCAGGGCC
