# Supplementary material for: Designer TALEs enable discovery of cell death-inducer genes
Source: Plant Physiol. 2024 May 9;195(4):2985–96. doi: 10.1093/plphys/kiae230 (PMC11288752; doi:10.1093/plphys/kiae230)
Supplement: kiae230_Supplementary_Data [file pp.01410.2023-s05.pdf › pp.01410.2023-s05.pdf]

Plant Physiology®

Designer TALEs enable discovery of cell death-inducer genes

Roxana Roeschlin, Sepideh Azad, René Grove, Ana Chuan, Lucila Garcia, Regina Niñoles, Facundo Uviedo, Liara Villalobos, Maria Massimino, Maria Marano, Jens Boch, and Jose Gadea

Review Timeline:

Submission Date:	24-Feb-2023
Editorial Decision:	29-Mar-2023
Resubmission Received:	04-Dec-2023
Editorial Decision:	16-Jan-2024
Revision Received:	11-Mar-2024
Accepted:	26-Mar-2024

29-Mar-2023

Dr. Jose Gadea
Institute for Molecular and Cellular Biology of Plants
ingeniero fausto elio s/n
Valencia 46022
Spain

RE: Designer TALEs enable discovery of cell death-inducer genes

Dear Dr. Jose Gadea:

Thank you for giving us the opportunity to review your manuscript for publication in the Plant Physiology. The reviewers have found the manuscript of interest, but have raised a number of concerns that will require your attention. As these concerns are of some gravity and will entail substantial changes to the manuscript, you are likely to need additional time to address them. I must therefore reject the manuscript in its present form. Nonetheless, if you are able to address the points raised in review, I would welcome your resubmission of a manuscript that incorporates your responses.

n particular, I direct your attention to the following comments for consideration in a resubmitted manuscript. A stronger connection between A4TA, as a TALE, and the targetted cell death genes must be provided with additional experiments. In addition, we recommend that the specified target plant genes be conclusively demonstrated to be the direct triggers of cell death.

Please, also be aware that reviewer 1 attached two documents with comments that also need to be addressed in a revised version.

Please note that any resubmission will be treated as a new entry to the system and is likely to go out for review.

If you do have any queries or concerns, you are welcome to contact me in the first instance.

Thank you again for allowing Plant Physiology to review your work. We look forward to hearing from you soon.

Sincerely,

Board of Editors, Plant Physiology

-- Reviewer comments:
Reviewer #1 (Comments for the Author):

The MS reports the identification of host genes that are reported to be induced by an unusually short TAL effector (7 RVD or 7 nucleotides specified by repeat region), here referred to as A4AT, and result hypersensitive-like response (HR) on Nicotiana benthamiana (Nb). By extending the repeat region in synthetic or designer TALEs, the author report narrowing in to specific genes that may be responsible for the HR. The work is interesting both in subject and approach. In the end, the authors surmise reasons for lack of short TALEs as maybe too non-specific and induce deleterious genes, whose expression and function interfere with infection. They also speculate that some genes could be used selected for R genes directed against full length TALEs in various disease complexes. Again, very interesting subject. Maybe akin to using defense genes such as NPR to elicit resistance with some manipulation. Nonetheless, the MS presents some questions to this reviewer that need to be addressed. No doubt the authors have identified some genes that, when ectopically expressed, result in an HR-like phenotype. However, the connection between A4TA, acting as a TALE, is questionable. A number of comments are in edited PDF. A few delineated here, in approximate order of seriousness, are as follows:

1. The phenotypes and population measurements of A4AT in Xcc are unusual for Xanthononas infectdions. No bacterial (0 CFU) at all at 24h with hrcV mutant? 10-2 log growth in EV strain to 10-12 log CGU in two days? This behavior is never seen in host infections. Nb is maybe a nonhost situation but rather remarkable results. The authors need to present a full growth curve from day 0 to 7-9 days out along with photos of leaf symptomology. If authors have documented the unusual behavior in previous publications, then include in Inftro.
2. Also, somewhat related to 1, the XopQ effector from Xcc is reported to trigger ROQ1-mediated HR in Nb (Schultink et al. f2017). The authors should comment on this. Is it possible that Xcc 8004 elicits a weak non-host response, and genes seen upon infection are just defense related, particularly PAT1, which has been observed before in defense responses.
3. Again, in relation to 1 and 2, no water-soaking is evident in Fig. 1A.
4. Could the authors generate a variety of short TALEs and find the same result? Seems to be their prediction.

5. The authors need to demonstrate that Ak4AT and some of the phenotype due to other dTALEs, like 26 or 27, for example, are dependent on NLS and/or activation domain. The use of Xanthomonas as a delivery vehicle would be nice also, ie would dTAL26 in Xcc trigger HR, or is only possible by agroinfection?
6. The presentation of all the host genes and dTALEs is very confusing. Little to no expression confirmations are performed. Some dTALEs work others do not. For example, in Fig. S9, the promoters of some genes are presented and gene NBlab11G19440.1, for example, is targeted by both dTAL26/55 and dTAL9.2, which give HR. At the same time, dTALG9.1 does not work (for HR). The gene is not in Table S1 or S2? Why are some genes in the tables and this one is not? Do the authors conclude 19440.1 is not a potential executor gene? Some how the authors need to make a more concise conclusion.
7. A presentation of the PAT1 promoter and potential binding sites for A4AT in fig. 2 would be nice. Again, is PAT1 chosen because it is induced in A4AT specific manner or is it just part of a defense response. What is qRTPCR of A4TA with and without activation domain, or, maybe just one of the RVD replacement variants. Again, it is not clear that PAT1 is TALE targeted al all in the WT strain.
8. The author give two hypothetical scenarios: 1) A specific toxic gene is induced or 2) complicated multigene expression results in HR. What about a Xa1 or BS4 like gene that recognizes TALEs or other effectors for that matter. Not all TALEs trigger BS4, so changing RVD in A4AT and losing HR does not necessarily show that it is DNA sequence specific loss of HR. Conformational changes may account for changes in NBS-LRR-type recognition of A4AT. At least, discuss this possibility.
9. In the end, the authors have identified some genes that can result in HR with delivered by agroinfection. This results may have been shown by others with other genes, not really aware of all the literature. Certainly. lesion mimics have been identified that result from aberrant expression. However, whether these genes are associated with the original HR due to the Xcc with TALE A4AT is not clear. Possible, but not demonstrated here.
10. Some similar notes and others in uploaded draft.

Reviewer #2 (Comments for the Author):

This manuscripts describes a systematic strategy to chase down a TAL effector target through the use of synthetic or 'designer' TAL effectors. Specifically, the paper is concerned with a very short TAL effector, PthA4^AT that triggers cell death in certain citrus varieties and N. benthamiana. Ultimately, the authors hit upon a few new cell death triggering genes, the strongest of which is a gibberellin-2-dioxigenase 6 type gene. The manuscript is well written, easy to follow and the data and figures are clear. The potential impact of this work is as follows: 1) demonstration of a generalizable approach of extending TALEs to identify their targets, 2) Identification of new plant cell death inducer genes, 3) Support for the idea that TAL induced executor genes arise from ectopic expression of endogenous triggers of PCD in plants. I have a few questions/comments that I suspect the authors can easily address related to the three impact points above.

1. The authors have achieved #1 very well and I suspect this approach to be adopted by future researchers.

2. For #2, I believe the authors are close, but need to do a few more experiments to demonstrate that the identified genes are indeed direct targets of the TAL and trigger cell death. In the case of the gibberellin-2-dioxigenase 6 (Figure 7), I believe only a single dTAL (or 2?) was pursued and no other confirmatory data is shown. It seems like a quick 35S promoter fusion, as was done in Figure 6, would be more conclusive. But even in this case, a downstream gene might still be the the actual trigger of cell death, correct?

3. With the above concern in mind, for #3, because the authors have not shown that the identified plant genes are the direct triggers of cell death, we do not yet know whether the presented data support this hypothesis.

4. Lines 123-124: wouldn't another initial hypothesis be recognition of the TAL effector by a protein that then triggers cell death? Similar to Bs4/avrBs4? If not, please explain why not. If this is a possible alternative hypothesis, then I think the authors need to address this alternative hypothesis throughout the manuscript.

5. Throughout the paper, I was unclear on when the authors were speculating about a possible number of predicted targets versus actual computational prediction. For example, line 153 indicates thousands of potential targets. Is this an estimate from one of the many TAL target prediction software programs? I think adding specific numbers of predicted targets, in a concise format, would be nice to see, especially for all the different dTALes. This type of analysis was done in a few places using PrediTALE and a lot of additional data is included in the supplemental tables for a few TALEs. I concise presentation of how much more specific the dTALes become with extension on C and N terminus, would strengthen the paper.

6. In lines 439-440: what is meant by 'natural host.' Is this the variety from which this strain was isolated?

7. In Figure 7, please list what the target genes are in the figure legend, instead of pointing to the supplemental table. It is ok to point to that table 'for more information about the genes' but the gene id's should be listed within the figure.

Minor comment: consider adding https://doi.org/10.1073/pnas.1620407114, 10.1094/MPMI-06-14-0161-R, and 10.1111/pbi.12677 to the references in lines 102-103 as other TAL susceptibility targets.

Reviewer #1 (Comments for the Author):

The MS reports the identification of host genes that are reported to be induced by an unusually short TAL effector (7 RVD or 7 nucleotides specified by repeat region), here referred to as A4AT, and result hypersensitive-like response (HR) on Nicotiana benthamiana (Nb). By extending the repeat region in synthetic or designer TALEs, the author report narrowing in to specific genes that may be responsible for the HR. The work is interesting both in subject and approach. In the end, the authors surmise reasons for lack of short TALEs as maybe too non-specific and induce deleterious genes, whose expression and function interfere with infection. They also speculate that some genes could be used selected for R genes directed against full length TALEs in various disease complexes. Again, very interesting subject. Maybe akin to using defense genes such as NPR to elicit resistance with some manipulation. Nonetheless, the MS presents some questions to this reviewer that need to be addressed. No doubt the authors have identified some genes that, when ectopically expressed, result in an HR-like phenotype. However, the connection between A4TA, acting as a TALE, is questionable. A number of comments are in edited PDF. A few delineated here, in approximate order of seriousness, are as follows:

We thank the reviewer for supporting our work and emphasizing our interesting subject and approach. We have now added experimental data and rewritten the manuscript according to this reviewer's suggestions.

1. The phenotypes and population measurements of A4AT in Xcc are unusual for Xanthononas infectdions. No bacterial (0 CFU) at all at 24h with hrcV mutant? 10^{-2} log growth in EV strain to 10^{-12} log CGU in two days? This behavior is never seen in host infections. Nb is maybe a nonhost situation but rather remarkable results. The authors need to present a full growth curve from day 0 to 7-9 days out along with photos of leaf symptomology. If authors have documented the unusual behavior in previous publications, then include in Inftro.

As the reviewer suggested, a more detailed growth curve of *Xcc8004* strains from day 0 to 9 has now been added to Figure 1. Although *N. benthamiana* is not a classical host of *Xcc*, our experimental setup is valid to see the impact

of PthA4^{AT} on bacterial proliferation. We show that the presence of PthA4^{AT} in *Xcc8004* results in a cell death response which severely reduces growth of *Xcc8004* in *N. benthamiana*. To test the need for nuclear localization of PthA4^{AT} for this cell death response, we transformed *Xcc8004* with a PthA4^{AT} mutant (mutNLS^{AT}), containing point mutations in the nuclear localization signals (Roeschlin et al., 2019). As Figure 1A shows, only *Xcc8004* harboring the non-mutated PthA4^{AT} triggers a cell death coupled with significant reduction of bacterial population. To reinforce the protection effect, we have now included findings in a second *N. benthamiana*-pathogen interaction. Tobacco necrosis virus A (TNVA) causes necrosis symptoms in *N. benthamiana* leaves. As shown in Figure 1B, the number of necrotic lesions after TNVA inoculation in leaves pre-infiltrated with Xcc expressing PthA4^{AT} was significantly reduced. This effect is also not observed using the PthA4^{AT} NLS mutant (mutNLS^{AT}) emphasizing that PthA4^{AT} is acting inside the nucleus to trigger cell death. A similar protection effect is observed in systemic leaves and shown in Figure 1C.

2. Also, somewhat related to 1, the XopQ effector from Xcc is reported to trigger ROQ1-mediated HR in Nb (Schultink et al. f2017). The authors should comment on this. Is it possible that Xcc 8004 elicits a weak non-host response, and genes seen upon infection are just defense related, particularly PAT1, which has been observed before in defense responses.

Indeed, the *Xanthomonas* effector HopQ triggers a resistance response in *N. benthamiana*, but *xopQ*-containing strains typically don't trigger an HR. The bacteria still grow in *N. benthamiana*, but they grow 10- to 100-fold better if *xopQ* is mutated. Similarly, transient *Agrobacterium*-mediated expression of *xopQ* does not trigger an HR, but only a mild chlorotic phenotype in *N. benthamiana* (Schultink et al., 2017). Taken together, we are aware that the *Xcc - N. benthamiana* interaction does not resemble a fully susceptible interaction, but is suitable to see additional resistance reactions.

Furthermore, the identification of PthA4^{AT} target genes in *N. benthamiana* was done using *Agrobacterium*-mediated infiltration. Therefore, *PAT1*-expression could not have been triggered by *Xcc* in our transcriptome analyses.

3. Again, in relation to 1 and 2, no water-soaking is evident in Fig. 1A.

The Figure 1A has been improved using new pictures to better display phenotypic responses 5 days post-inoculation.

4. Could the authors generate a variety of short TALEs and find the same result? Seems to be their prediction.

We thank the reviewer for this comment. We have been discussing this issue. As we demonstrated in section 2 in Results (PthA4^{AT}-mediated cell death is dependent on specific RVDs),modifying the RVDs in this short TALE disrupts the induction of cell death. This indicates that not every short TALE causes the same results, and the effect we study here, therefore, is specific of PthA4^{AT}.

The following paragraph in the discussion where this scenario was suggested (former line 422) has been removed for clarity:

REMOVED: *"The ability of PthA4AT to trigger cell death in N. benthamiana via activation of several cell death ex-R genes could explain why the existence of short TALEs retaining the ability to activate gene expression is so limited in nature. Natural TALEs contain a mean of 17.5 RVDs and this number coincides with the maximum specificity of TALEs, indicating that this length has been selected by evolution to achieve high target specificity (Boch and Bonas, 2010; Rinaldi et al., 2017). Shorter TALEs might be too prone to activate "hidden" executor genes in a plant genome, which would eventually ruin the infection process, and bacteria carrying those TALEs will be counterselected"*

5. The authors need to demonstrate that Ak4AT and some of the phenotype due to other dTALEs, like 26 or 27, for example, are dependent on NLS and/or activation domain. The use of Xanthomonas as a delivery vehicle would be nice also, ie would dTAL26 in Xcc trigger HR, or is only possible by agroinfection?

We have added to the Introduction previous results from our group (Roeschlin et al, 2019) which demonstrate that the cell death phenotype of PthA4^{AT} is dependent on NLS and activation domain. New manuscript lines 97-102:

"Disrupting the three NLS of PthA4AT by point mutations impairs the development of this response, indicating that nuclear localization is required.

Moreover, PthA4AT is able to mediate transcriptional activation, and the HR is greatly attenuated when the C-terminal domain containing the activation domain is removed, pointing to a mechanism for PthA4AT perception operating both in Citrus limon and N. benthamiana based on host gene expression (Roeschlin et al., 2019)"

We have reinforced this also in the first section of Results, showing that PthA4^{AT} renders protection to pathogens but the mutated version in NLS does not (lines 125-133: "*Similarly to Citrus (Roeschlin et al., 2019), PthA4AT is translocated into the N. benthamiana nucleus (Supplemental Figure S1), and the arrest of bacterial progression was not observed in leaves inoculated with a Xcc8004 expressing the PthA4AT derivative mutated in the NLSs (mutNLSAT) (Figure 1A).Similar results were obtained using a different plant-pathogen interaction. Agroinfiltration of PthA4AT into one half of the N. benthamiana leaf and subsequent rub inoculation with tobacco necrosis virus A (TNVA) into the neighbouring half, significantly reduced the number of virus-induced necrotic lesions, whereas using the mutNLSAT variant did not (Figure 1B). Furthermore, the number of distal leaves with necrosis symptoms also decreased in PthA4AT-infiltrated plants relative to the controls (Figure 1B).*

TNVA protection has been shown now also for two PthA4^{AT} derivates, dTAL1 and dTALE26 (supplemental figures S2 and S6). In these experiments, *Xanthomonas* was used as delivery vehicle, as suggested by the reviewer.

6. The presentation of all the host genes and dTALEs is very confusing. Little to no expression confirmations are performed. Some dTALEs work others do not. For example, in Fig. S9, the promoters of some genes are presented and gene NBlab11G19440.1, for example, is targeted by both dTAL26/55 and dTAL9.2, which give HR. At the same time, dTALG9.1 does not work (for HR). The gene is not in Table S1 or S2? Why are some genes in the tables and this one is not? Do the authors conclude 19440.1 is not a potential executor gene? Some how the authors need to make a more concise conclusion.

We have now simplified and streamlined this point. Gene-specific dTALEs were designed in the laboratory as tools to activate target gene expression. Some of them can be successful in that purpose and some others cannot. We have modified the manuscript in these aspects:

a) In the previous version of the manuscript, we described several potential cell death inducer genes and the corresponding dTALEs we designed although several did not trigger an HR. Now, we have omitted all the information concerning target candidates and the corresponding dTALEs which were not able to trigger HR and were probably no cell death inducer genes. In this resubmission, we maintain only information concerning one clear target gene: NBlab07G09180. Accordingly, former Supplemental Fig S9 has been removed, and the graphical representation of the promoter of gene NBlab07G09180 is now in Figure 6.

b) In panel A in Figure 6, we show all the different dTALEs we designed to activate this gene, and in panel B we show how many of them were successful in activating the gene. Expression by qRT-PCR is also shown for all dTALEs.

We conclude that NBlab18G05420 (PAT1) and NBlab07G09180 (NEC3) are *bona fide* cell-death inducer genes.

7. A presentation of the PAT1 promoter and potential binding sites for A4AT in fig. 2 would be nice. Again, is PAT1 chosen because it is induced in A4AT specific manner or is it just part of a defense response. What is qRTPCR of A4TA with and without activation domain, or, maybe just one of the RVD replacement variants. Again, it is not clear that PAT1 is TALE targeted al all in the WT strain.

The $PthA4^{AT}$/dTal26-binding site in the *PAT1* promoter is shown in Figure 4A.

This gene was selected based on promoter sequence and gene expression criteria. PAT1 is induced in a dTal26-specific manner. It was selected based on binding and expression criteria and further studied by overexpression. That the gene is also induced in other interactions in plant defense had no influence at this point. The PthA4-AT binding site is overlapping with dTal26's, so there is no indication that the gene will not be activated by $PthA4^{AT}$ if it is activated by dTal26. In fact, this has been shown in Figure 4 using dTal1 which is equivalent to $PthA4^{AT}$. The requirement of the activation domain was already demonstrated for PthA4-AT in a previous manuscript (Roeschlin et al, 2019).

8. The author give two hypothetical scenarios: 1) A specific toxic gene is

induced or 2) complicated multigene expression results in HR. What about a Xa1 or BS4 like gene that recognizes TALEs or other effectors for that matter. Not all TALEs trigger BS4, so changing RVD in A4AT and losing HR does not necessarily show that it is DNA sequence specific loss of HR. Conformational changes may account for changes in NBS-LRR-type recognition of A4AT. At least, discuss this possibility.

The PthA4^{AT}-HR does not occur if either the NLS or the activation domain are mutated (see Fig. 1 and Roeschlin et al., 2019). This strongly suggests that gene activation and the subsequent activity of the target gene(s) is the cause of HR. An NLR-based resistance reaction would not be compromised by these mutations. We now add this point to the discussion (lines 290-293).: "Moreover, the possibility of PthA4A^{T-}mediated cell death being triggered after recognition of the effector in the cytoplasm in a Bs4-like matter is discarded, as disrupting the three NLSs or removing the activation domain impairs the development of this response.

9. In the end, the authors have identified some genes that can result in HR with delivered by agroinfection. This results may have been shown by others with other genes, not really aware of all the literature. Certainly. lesion mimics have been identified that result from aberrant expression. However, whether these genes are associated with the original HR due to the Xcc with TALE A4AT is not clear. Possible, but not demonstrated here.

We agree with this reviewer that some genes whose ectopic expression cause cell death were discovered earlier using overexpression strategies. Some of these strategies are mentioned in the Discussion (Nasir et al., 2005; Coemans et al., 2008). In our case, starting from a natural effector causing HR, we went downstream to identify genes which are activated by this effector. Finally, designer dTALEs for these specific genes were demonstrated to activate HR similar to the original effector. This is typically considered sufficient evidence to identify a gene as a target of a TALE which is responsible for a given phenotype. This has been a common strategy used to discover TALE-dependent genes, for example in:

Streubel et al, 2013. Five phylogenetically close rice SWEET genes confer TAL effector-mediated susceptibility to Xanthomonas oryzae pv. oryzae. New Phytol. 2013 Nov;200(3):808-819

Hu Y, Zhang J, Jia H, Sosso D, Li T, Frommer WB, Yang B, White FF, Wang N, Jones JB. Lateral organ boundaries 1 is a disease susceptibility gene for citrus bacterial canker disease. Proc Natl Acad Sci U S A. 2014 Jan 28;111(4):E521-9

Hummel et al, 2017. A transcription activator-like effector from Xanthomonas oryzae pv. oryzicola elicits dose-dependent resistance in rice. Mol Plant Pathol. 2017 Jan;18(1):55-66

10. Some similar notes and others in uploaded draft.

We answer the different notes below:

In main text:

Remove first paragraph. PCD is not really addressed in MS. No measures of PCD are conducted. All responses are assumed to be hypersensitive reaction, which is reported to be form of PCD and ref to this is sufficient. Maybe report more details about PthA4AT.

As suggested, we have removed this paragraph, as well as the last one, from the introduction. We have provided more details about $PthA4^{AT}$.

Three scenarios. Should include an BS4-type resistance.

This third scenario was discarded based on the requirements of NLS and activation domain (see Fig. 1 and Roeschlin et al, 2019). This hopefully answers further related concerns from the reviewer on the same subject.

What is the evidence from literature that such aberrant transcription results in cell death?

We though that this could be possibility, but there is no bibliography to support that this is the case. We have now omitted from the text this possibility by removing the sentence: "*PthA4AT could mediate a broad and aberrant activation of multiple genes, thereby disrupting the standard transcriptomic program in the cell, eventually resulting in cell death*".

Why NN or NH?

The recognition code of every RVD is now stated at the beginning of the section "PthA4AT-mediated cell death is dependent on specific RVDs". In Line 140: "*The four most common RVDs in nature (NI, HD, NN and NG) tend to specify bases A, C, G/A and T, respectively. Less common RVDs such as N* can target C/ T, and NH achieve guanine-specific recognition. NS, however, can target any of the four bases.*" Moreover, the difference between NN and NH

is further stated in line 177: "*with NN in its last position substituted by NH, to confer better specificity for G*".

Why the switch to agro-infection?

We have been using infiltration of Agrobacterium along the whole study, to assure that downstream effects are caused only by the effector PthA4^{AT} or other TALEs and not by Xanthomonas in general.

Maybe discuss RoqQ/XopQ story since Xcc reproted to have XopQ that triggers resistance in Nb.

The protection effect is not observed in wild-type Xcc8004, which contains XopQ, nor using PthA4^{AT} with mutated NLS (see Figure1). This excludes the possibility that resistance is mediated by XopQ.

Figure 2. Panel D.

How is it known that the points of GFP are nuclei?

We have repeated the experiment using DAPI staining and merging the images.

Is this a verb?

We did not use "Agroinfiltrated" in this new version. In contrast, "Agroinfiltration" is a word that is commonly used in the literature.

Former Figure 3. Panel A.

Include RVDs for PthA4AT. Are they identical to dTal1? Why not just put RVD instead of colar? Could be challenge for color blind readers and just adds another layer code to frustrate all readers.

The PthA4^{AT} RVDs are the same as the dTal1 RVDs, as indicated in the text. In Figure 2, this is noted in Panel A. For the rest of the figures, the dTal1 RVDs are always shown as a reference. We agree that the color code can be problematic for some readers and, accordingly, have changed all figures to avoid color-codes and support color blind readers.

Former Figure 5

What is C-? Water, PTHA4AT?

C- is infiltration medium which is now stated in the figure legend.

Former Figure 6

Please highlight the potential PTH4AT upa binding sites.

It is highlighted in panel A (dTal26) in a box and also indicated in the legend.

What is WT here? In Panel E

This is now indicated (now Figure 4): Control, -infiltration medium.

In supplementary data:

former Suplementary Figure S2:

WT Xcc needed

This experiment does not use Xcc, but infiltration by *Agrobacterium*, as the legend indicates. The experiment and panel have been rearranged adding a control with infiltration medium, which shows no HR.

Former Supplemenmtal Figure S4:

Is C- buffer?

The C- is infiltration medium and has now been detailed in the legend.

What host gene(s) is targeted by 27? And 45-48?

Likely many genes targeted by dTal26 will also be targeted by 27 and 45-48, as sequence differences are minimal, and TALEs accept some mismatches. Concerning the cell death inducer genes, we presume that they are the same ones. Identifying targets of other TALEs different than dTal26, dTal50 and dTal55 is not approached further in this study.

Former Supplemental Figure S9:

This annotation not in Table S1 or S2. Or bolded.

The gene is in Table S2

Was there suppose to be a table here? Or just supplemental stand alone table file?

Again, maybe missed something here. Is there supose to be table here?

The tables are uploaded as separate files.

Reviewer #2 (Comments for the Author):

This manuscripts describes a systematic strategy to chase down a TAL effector target through the use of synthetic or 'designer' TAL effectors. Specifically, the paper is concerned with a very short TAL effector, PthA4^AT that triggers cell death in certain citrus varieties and N. benthamiana. Ultimately, the authors hit upon a few new cell death triggering genes, the strongest of which is a gibberellin-2-dioxigenase 6 type gene. The manuscript is well written, easy to follow and the data and figures are clear. The potential impact of this work is as follows: 1) demonstration of a generalizable approach of extending TALes to identify their targets, 2) Identification of new plant cell death inducer genes, 3) Support for the idea that TAL induced executor genes arise from ectopic expression of endogenous triggers of PCD in plants. I have a few questions/comments that I suspect the authors can easily address related to the three impact points above.

1. The authors have achieved #1 very well and I suspect this approach to be adopted by future researchers.

We thank the reviewer for this positive comment and for supporting our approach.

2. For #2, I believe the authors are close, but need to do a few more experiments to demonstrate that the identified genes are indeed direct targets of the TAL and trigger cell death. In the case of the gibberellin-2-dioxigenase 6 (Figure 7), I believe only a single dTAL (or 2?) was pursued and no other confirmatory data is shown. It seems like a quick 35S promoter fusion, as was done in Figure 6, would be more conclusive. But even in this case, a downstream gene might still be the the actual trigger of cell death, correct?

We have extended the gene-specific dTALE strategy by designing 8 dTALEs targeting the NBlab11G19440 gene, and 6 dTALEs targeting the NBlab07G09180 gene. Also, as suggested, we generated 35S promoter fusions for these candidates. This more elaborate experimental design allowed us to confirm NBlab07G09180 as a bona fide cell death inducer gene, besides PAT1.

In contrast, gene NBlab11G19440 gave variable results, as its expression shows a strong HR in many cases, but no HR in others. We believe that additional environmental factors might influence this cell death reaction. For the sake of clarity, we focused on the unambiguous NBlab07G09180 candidate in this manuscript and omitted the variable NBlab11G19440 gene.

The reviewer is correct that it is possible that not only the targets described here, but also downstream genes contribute to the HR. It will be interesting in future research to understand which signals are integrated by the cell to eventually commit to cell death.

3. With the above concern in mind, for #3, because the authors have not shown that the identified plant genes are the direct triggers of cell death, we do not yet know whether the presented data support this hypothesis.

What we report here is that the genes identified are expressed directly via the TALE. Also, expressing the genes by themselves generates HR. Therefore, these genes are causal agents of cell death. Whether this includes additional downstream genes and cellular processes is unknown. Albeit, this is also the case for other ex-R resistance genes (Bs3, Xa7, Xa10, Xa23, etc.). We believe that our addition of novel cell death inducer genes opens new avenues to track down the cellular signals for cell death.

Nowack et al, 2022 discuss the possibility of executor genes arising from ectopic expression of endogenous triggers of HR. What they propose is that the initial target for the final effect (HR) is the ex-R, although the HR itself can be generated by different downstream genes. We believe that this way of expressing the concept of "cell death-inducer" or "ex-R" which we also follow in this manuscript is adequate and assumed by readers.

4. Lines 123-124: wouldn't another initial hypothesis be recognition of the TAL effector by a protein that then triggers cell death? Similar to Bs4/avrBs4?

If not, please explain why not. If this is a possible alternative hypothesis, then I think the authors need to address this alternative hypothesis throughout the manuscript.

Because the HR triggered by $PthA4^{AT}$ is dependent on the NLS and activation domain, we believe that NLR proteins like Bs4 are not involved. The Bs4 recognition is not compromised if NLS or AD is mutated. We now add a section to the discussion to clarify this point to the reader.

[In this resubmission, we have explained more in detail all this so that the concern of a putative Bs4/avrBs4-like recognition event is discarded.] New manuscript lines 97-102: "*Disrupting the three NLS of PthA4AT by point mutations impairs the development of this response, indicating that nuclear localization is required. Moreover, PthA4AT is able to mediate transcriptional activation, and the HR is greatly attenuated when the C-terminal domain containing the activation domain is removed, pointing to a mechanism for PthA4AT perception operating both in Citrus limon and N. benthamiana based on host gene expression (Roeschlin et al., 2019)*

Also lines 125-133 (in Results) and again in the Discussion (lines 290-293: *"Moreover, the possibility of PthA4AT-mediated cell death being triggered after recognition of the effector in the cytoplasm in a Bs4-like matter is discarded, as disrupting the three NLSs or removing the activation domain impairs the development of this response."*

5. Throughout the paper, I was unclear on when the authors were speculating about a possible number of predicted targets versus actual computational prediction. For example, line 153 indicates thousands of potential targets. Is this an estimate from one of the many TAL target prediction software programs? I think adding specific numbers of predicted targets, in a concise format, would be nice to see, especially for all the different dTALes. This type of analysis was done in a few places using PrediTALE and a lot of additional data is included in the supplemental tables for a few TALEs. I concise presentation of how much more specific the dTALes become with extension on C and N terminus, would strengthen the paper.

We agree with the reviewer that the first comment, "thousand of potential targets" was a colloquial way to say that such a short sequence can be predicted to bind to many targets according to probability calculations. We have deleted this sentence from the paragraph.

On the other hand, adding specific numbers of potential targets to the different dTALEs with different length is very difficult. The prediction software PrediTALE identifies possible target sequences in promoter regions of the genome and ranks them according to their probability score. We did notice that these probabilities (p-values) get lower with increasing length of the dTALE which indicates that the software gives them a better chance to bind the corresponding target sequence, likely because longer TALEs have less off-targets. Nevertheless, the algorithm is not suitable to give absolute values for targets that can be compared across TALEs with different numbers of repeats. We must admit that we are currently not able to give a bioinformatic solution that is scientifically solid to the reviewer's request.

6. In lines 439-440: what is meant by 'natural host.' Is this the variety from which this strain was isolated?

Yes, we have modified the sentence accordingly (now line 362).

7. In Figure 7, please list what the target genes are in the figure legend, instead of pointing to the supplemental table. It is ok to point to that table 'for more information about the genes' but the gene id's should be listed within the figure.

We now focus in this section on one clear target gene (NEC3) and this gene is now shown in Figure 6 (former Figure 7). The supplemental table is omitted to streamline the manuscript.

Minor comment: consider adding https://doi.org/10.1073/pnas.1620407114, 10.1094/MPMI-06-14-0161-R, and 10.1111/pbi.12677 to the references in lines 102-103 as other TAL susceptibility targets.

We thank the reviewer for this information and have now added Schwartz et al 2017 and Cohn et al, 2014 to the references as they use similar approaches to discover TALE-susceptibility genes.

16-Jan-2024

Dr. Jose Gadea
Institute for Molecular and Cellular Biology of Plants
ingeniero fausto elio s/n
Valencia 46022
Spain

RE: Designer TALEs enable discovery of cell death-inducer genes

Dear Dr. Gadea:

We have received reviews of your manuscript, which you may view at the end of this letter. On the basis of the advice received and my own evaluation, I believe that, pending revision, this article may ultimately be acceptable for publication in Plant Physiology.

In addition to the comments of Reviewer 3, I suggest adding the dTALE26 cell death testing and the corresponding plant gene screening in Citrus limon as additional proof of concept.

When you are ready to submit your revision, click the below link and find the red arrow under your Author Tasks heading for this revision submission.

If you want to visit this version of the manuscript, you may click the link below and then click on the folder that reads "Post Decision Manuscripts".

Link Not Available

When uploading the revised version of this article, please be sure to include as complete an answer as possible to the referees' criticisms during the first round of reviews in the "response to reviewers" section.

Plant Physiology considers a revision for up to 60 days after a manuscript has been provisionally accepted; limited extension of this timeframe is at the discretion of the Monitoring Editor. A reminder notice will be sent to you 2 weeks before the deadline, at which time you may choose to contact the editorial office to request an extension; if so, please provide a justification for your request. After the expiration of this deadline, the article would need to be submitted as a resubmission and would be handled as such.

Thank you very much for giving us an opportunity to review this work. I look forward to receiving the next version.

Sincerely,

Board of Editors
Plant Physiology

Want to add this revision deadline to your calendar? Click below!

-- Reviewer comments:
Reviewer #2 (Comments for the Author):

The authors have addressed most of my concerns and the manuscript is improved. On a few points, the authors were unable to address my concerns but these were not critical points. I thanks the authors for doing a nice job with their responses.

Reviewer #3 (Comments for the Author):

The manuscript characterized the cell death (HR-like) phenotype in Nicotiana benthamiana induced by a short TALE (PthA4AT) identified from Xanthomonas citri AT. It described the RVD-specific nature of this TALE triggered cell death and its potential of

disease progress inhibiting effects. To identify the plant gene targets of PthA4AT that induce the cell death, a step-wise extension of the RVD domain was performed to screen for specific longer target sequences. Using the extended 10-14 RVD constructs and RNA-seq, a few candidate plant genes were identified (PAT1 and NEC3), while results indicate more target genes in play. The manuscript presented a systematic strategy for narrowing down plant targets for TALEs with distinct symptoms using designer TALEs, which could be useful especially for short TALEs.
Meanwhile, a few issues and questions arose during the review. Hopefully addressing these would serve to make the manuscript clearer and more convincing.
Major comments and questions:
1. P6 L147-148 and Supplement Fig S2 B: The number of local lesions per cm2 in the dTal1 treated leaves are significantly more than that in the control. This is not the similar phenomenon observed with the experiment of PthA4AT (Fig 1 B showing reduced number of local lesions). Please describe/clarify accordingly in the text.
2. What were the reasons for dTal26 been selected for further extension and target screening while dTal21 or dTal22 were dropped? Based on Fig 3 data, it seems the derivative dTALEs with HD at the 9th position even have higher specificity at the 10th RVD. Wouldn't they yield interesting targets?
3. Fig 4 B and D. To better demonstrate and compare the effect of dTALEs and the controls, it would be more convincing if symptoms of the negative control and 35S::PAT1 were shown together on the same leaf with the PAT1-dTALEs constructs.
4. P8 L247-249. Not sure the mention of low expression of 35S::PAT1 at 36hpi supports the statement that "PAT1 expression levels alone only cause a delayed cell death". It seems to indicate that the 35S::PAT1 construct itself is not as effective as the dTALEs. Also, this whole paragraph presents several pieces information and evidence but could use a bit re-organization to address the main finding.
5. Cannot find .xlsx files for supplement table S1 and S2 in the submitted files.
Minor issues:
1. Fig 3 C. Please consider adding statistical analysis for the conductivity.
2. Fig 6 B. Missing hpi label for when the cell death symptoms were recorded.
3. Fig S3. The conductivity measurements may be convincing enough, but it may be better to include dTal9 symptom at 72dpi in the panel B, to rule out a weaker cell death.
4. P12 L367 What's definition of a "short" TALE here to support this statement?
5. P13 L417-418. Not clear what was sampled exactly for each replicate and repeat. Consider checking for similar expressions in the Methods section.

Reviewer #3 (Comments for the Author):

The manuscript characterized the cell death (HR-like) phenotype in Nicotiana benthamiana induced by a short TALE (PthA4AT) identified from Xanthomonas citri AT. It described the RVD-specific nature of this TALE triggered cell death and its potential of disease progress inhibiting effects. To identify the plant gene targets of PthA4AT that induce the cell death, a step-wise extension of the RVD domain was performed to screen for specific longer target sequences. Using the extended 10-14 RVD constructs and RNA-seq, a few candidate plant genes were identified (PAT1 and NEC3), while results indicate more target genes in play. The manuscript presented a systematic strategy for narrowing down plant targets for TALEs with distinct symptoms using designer TALEs, which could be useful especially for short TALEs. Meanwhile, a few issues and questions arose during the review.

Hopefully addressing these would serve to make the manuscript clearer and more convincing.

Major comments and questions:

1. P6 L147-148 and Supplement Fig S2 B: The number of local lesions per cm2 in the dTal1 treated leaves are significantly more than that in the control. This is not the similar phenomenon observed with the experiment of PthA4AT (Fig 1 B showing reduced number of local lesions). Please describe/clarify accordingly in the text.

We are sorry about that. The images did not match the corresponding data (they were interchanged). The Fig S2B has been updated to accurately reflect the findings.

2. What were the reasons for dTal26 been selected for further extension and target screening while dTal21 or dTal22 were dropped? Based on Fig 3 data, it seems the derivative dTALEs with HD at the 9th position even have higher specificity at the 10th RVD. Wouldn't they yield interesting targets?

dTal26 was chosen for stepwise extension and target identification as an example of a dTal showing HR whose targets could be identified using our strategy, but certainly dTal21 or dTal22 could have been also selected for this purpose. dTal25, with NH in the 9th position, also showed HR, so any of the two “pairs” were good candidates. Whether the other branch (starting in dTal21) would be finally yielding the same or other interesting targets is difficult to know. Given the similarity of all dTals and the way of action of TALE effectors in terms of mismatch acceptance, we speculate that the same targets would be bound by dTal21 derivatives with altered affinities, but this assertion is speculative at this moment.

3. Fig 4 B and D. To better demonstrate and compare the effect of dTALEs and the controls, it would be more convincing if symptoms of the negative control and 35S::PAT1 were shown together on the same leaf with the PAT1-dTALEs constructs.

The slight differences in inoculation procedures make direct biological comparison challenging (e.g., p19 silencing suppressor strain is present in 35S constructs but not in dTALEs assays). To avoid misinterpretation, we maintain separate panels for each experiment in Fig 4.

However, both assays were repeated on the same leaf as requested by the Reviewer 3, yielding similar results to those in panels B and D (Figure 1). The 35S construct induces more cell death 6 days post-inoculation. Regardless, the crucial point is that cell death occurs when PAT1 is activated. We include results from the new assay for Reviewer consideration but maintain Fig 4 panels B and D unchanged.

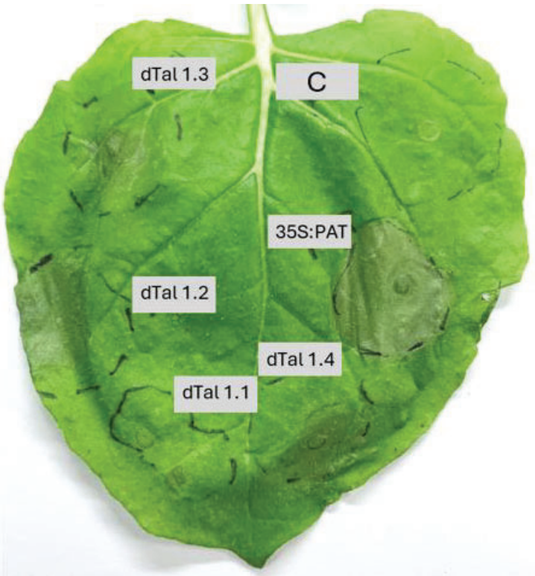

Figure 1. Cell death response in *N. benthamiana* leaves infiltrated via *Agrobacterium* containing the *PAT1* cDNA under the control of the CaMV35S promoter and *PAT1*-dTALEs constructs. Picture was taken at 6 days post inoculation (dpi). C: infiltration medium.

4. P8 L247-249. Not sure the mention of low expression of 35S::PAT1 at 36hpi supports the statement that "PAT1 expression levels alone only cause a delayed cell death". It seems to indicate that the 35S::PAT1 construct itself is not as effective as the dTALEs. Also, this whole paragraph presents several pieces information and evidence but could use a bit re-organization to address the main finding.

We have removed this statement from the paragraph on P8L247-249 of the Results. We would not like to state that the 35S:PAT1 is less effective than the dTALEs, because when 35S-mediated expression of the transgene increases over time, the phenotypic effects seem stronger than that obtained by using the dTALEs.

5. Cannot find .xlsx files for supplement table S1 and S2 in the submitted files.

The .xlsx files for Supplemental tables S1 and S2 are included in the supplementary material attached to the submission application. Please check the supplementary material section for

access to these files. If you encounter any issues accessing them, please let us know, and we will ensure they are provided promptly.

Minor issues:

1. Fig 3 C. Please consider adding statistical analysis for the conductivity.

Statistical analysis has been applied to the conductivity measurements, including the data presented in Fig 3C, as well as others conductivity measurements (Fig. 2C, 6C, S3C).

2. Fig 6 B. Missing hpi label for when the cell death symptoms were recorded.

This data has been added

3. Fig S3. The conductivity measurements may be convincing enough, but it may be better to include dTal9 symptom at 72dpi in the panel B, to rule out a weaker cell death.

In response to Reviewer suggestion, we have included a new panel in Figure S3 showing the symptoms of dTal9 at 72 hpi. This addition aims to provide further evidence and rule out any possibility of weaker cell death at this time point.

4. P12 L367 What's definition of a "short" TALE here to support this statement?

We have clarified the issue by adding the number of repeats of $PthA4^{AT}$. The ability to activate gene expression of 7.5-repeats TALEs was demonstrated by Boch et al in 2009. The ability of this particular TALE to activate gene expression was demonstrated by Roeschin et al, 2019. Concerning the possibility that this short TALE originated by recombination, we have removed this comment on P12 L367, as it is not pertinent to this discussion.

5. P13 L417-418. Not clear what was sampled exactly for each replicate and repeat. Consider checking for similar expressions in the Methods section.

The term “repeat” in the Cloning Strategy section refers to the modules needed for reconstructing every dTALE, according to Geißler et al., 2011. “Repeat” meaning the region comprising the 33 amino acids. The term replicate is used in a different context, meaning the number of times an experiment is repeated. What is sampled for each replicate in every section is now consistently explained.

We have improved Materials and Methods section and clarified this issue in P13 L411, P13 L431, P14 L434-435, P14 L439-440, P14 L447-449.

26-Mar-2024
Dr. Jose Gadea
Institute for Molecular and Cellular Biology of Plants
ingeniero fausto elio s/n
Valencia 46022
Spain

MSID: PP2023-RA-01410DR1
MS TITLE: Designer TALEs enable discovery of cell death-inducer genes

Dear Dr. Jose Gadea:

I am pleased to inform you that your revised manuscript "Designer TALEs enable discovery of cell death-inducer genes" has been accepted for publication in Plant Physiology. Your manuscript will be checked for consistency with journal formatting and image standards by one of our Science Editors, who will contact you shortly. They may have a number of queries and suggestions for you. Please keep in mind that they work to increase the online visibility of your article and its attraction for readers from the widest of backgrounds. You will have the opportunity to review any revisions, and I hope you will work constructively with them to get the most out of your research. You will also have the opportunity to review all changes when you receive the author proofs for your article.

Once validated for publication, your article will be sent to a Science Editor for a final review of your manuscript and source files. After your final files are uploaded, your article will appear in the next available issue and the online Preview version of your article will be posted after your signed Copyright and Checklist forms and final source files are received.

Please be sure to include the following in your manuscript file:
Harvard style citations
List of all author contributions and funding information at the beginning of manuscript after the titles and author list.
List all supplemental materials associated with the article, with titles, at the end of the ms, before the references and acknowledgments section.
Complete Figure Legends for all figures appearing in the article listed before the Literature Cited.

Thank you and congratulations on your Plant Physiology paper!

Sincerely,
Board of Editors, Plant Physiology

=====
FIRST AUTHOR PROFILES
=====

We recognize the hard work that goes into being First Author of a Plant Physiology paper by publishing a "First Author Feature" for each First Author alongside your paper.

If the first author(s) would like to submit information for a feature on Facebook and Twitter, fill out the form by clicking on the link below:

Plant Physiology First Author Feature Form: https://forms.office.com/r/ZBCGZfhm8F
If you cannot access the form through the link above, please contact Rachel at rbelsky@aspb.org.

To upload an image of yourself to accompany your profile, please put your photo in the correct journal folder at the link below. You must label your image: Author First Name Last Name Article Title.

First Author Profile Image Dropbox: https://www.dropbox.com/scl/fo/xz029p7qjr2jml2rp2kmq/h?dl=0&rlkey=6fkxcaqmxx35ap152b2osy1fz

=====

IMPORTANT REMINDER: PEER REVIEW REPORTS
=====

If you opted to publish a peer review report along with your article during the original submission process, it will be prepared by the editorial staff and publicly posted with your manuscript, inside the zip file that contains any other supplemental material. As a reminder, the peer review report is a public record of all comments from editors and reviewers, as well as your prior responses, as you received them in the decision letters for each draft of your manuscript. If you agreed to publish this report and have changed your mind, or are not sure if you selected this option, please contact the editorial office as soon as possible before signing the license agreement from our publisher.

-- Reviewer comments:
Reviewer #4 (Comments for the Author):

The manuscript is well written with clear methods. Authors have addressed previously raised issues.

The manuscript aim to be a proof of concept for a new strategy to extend dTALE and screen for host targets. While the TALE was identified from Citrus limon, it causes cell death on both C. limon and N. benthamiana. All the dTALE extention, testing and target screening in this MS were done in N. benthamiana. Whether the extended dTALEs could cause the same cell death symptoms as PthA4AT, and identify corresponding plant genes in the citrus host is unknown.

It is recommended to add dTALE26 cell death testing and the corresponding plant gene screening in Citrus limon to suffice a proof of concept. Additional testing may take considerable time, please feel free to discuss if the authors find it unnecessary.
